# Identifying a Novel Endoplasmic Reticulum-Related Prognostic Model for Hepatocellular Carcinomas

**DOI:** 10.1155/2022/8248355

**Published:** 2022-07-22

**Authors:** Fei Ding, Jinping Li, Yong Zhang, Chengang Wang, Yonghua Yu

**Affiliations:** ^1^Shandong University Cancer Center, Jinan, Shandong, China; ^2^Department of Radiation Oncology and Shandong Provincial Key Laboratory of Radiation Oncology, Shandong Cancer Hospital and Institute, Shandong First Medical University and Shandong Academy of Medical Sciences, Jinan, Shandong, China; ^3^Research Unit of Radiation Oncology, Chinese Academy of Medical Sciences, Jinan, Shandong, China; ^4^Department of Oncology, Zibo First Hospital, Zibo, China; ^5^Department of Public Health, Zibo First Hospital, Zibo, China

## Abstract

From the standpoint of the ER (endoplasmic reticulum), we were interested in identifying hub genes that impact clinical prognosis for HCC (hepatocellular carcinoma) patients and developing an ER-related prognostic model. Using TCGA-LIHC (The Cancer Genome Atlas-Liver Hepatocellular Carcinoma) and GSE14520 datasets, we conducted a series of analyses, which included differential gene screening, clinical prognostic analysis, Lasso regression, nomogram prediction, tumour clustering, gene functional enrichment, and tumour infiltration of immune cells. Following our screening for ER-related genes (*n* = 1975), we conducted a Lasso regression model to obtain five hub genes, KPNA2, FMO3, SPP1, KIF2C, and LPCAT1, using TCGA-LIHC as a training set. According to risk scores, HCC samples within either the TCGG-LIHC or GSE14520 cohort were categorized into high- and low-risk groups. Compared to the high-risk group of HCC patients, patients in the low-risk group had a better prognosis of OS (overall survival) or RFS (relapse-free survival). For TCGA-LIHC training set, with the factors of risk score, stage, age, and sex, we plotted a nomogram for 1-, 3-, and 5-year survival predictions. Our model demonstrated better clinical validity in both TCGA-LIHC and GSE14520 cohorts. Additionally, events related to biological enzyme activity, biological metabolic processes, or the cell cycle were associated with the prognostic risk of ER. Furthermore, two HCC prognosis-associated tumour clusters were identified by ER hub gene-based consensus clustering. Our findings indicated a link between ER prognostic signature-related high/low risk and tumour infiltration levels of several immune cells, such as “macrophages M2/M0” and “regulatory T cells (Tregs).” Overall, we developed a novel ER-related clinical prognostic model for HCC patients.

## 1. Introduction

As the primary type of liver cancer, HCC has an increasing incidence and high mortality rate worldwide [1, 2]. The occurrence and progression of HCC involve the disruption of multiple organelle functions within cells, including the undesirable stimulation of the endoplasmic reticulum (ER) and mitochondria [3]. Various genes may play roles in the pathogenesis of human diseases, including cancers, by affecting the normal physiological functions of specific organelles and maintaining cellular homeostasis [4, 5]. By utilizing different machine learning approaches, a series of survival prediction models targeting specific biological events were developed [6–11]. For instance, Lasso (least absolute shrinkage and selection operator) regression was applied to build several prognostic models for HCC patients targeting ferroptosis [8], reactive oxygen species [9], amino acid metabolism [11], or the tumour microenvironment [7]. In this study, we first constructed an HCC prognostic model from the perspective of ER using an integrated modelling strategy (differentially expressed genes, univariate/multivariate Cox regression, Lasso regression, and nomogram prediction). ER-associated gene expression datasets and the detailed clinicopathological parameters of HCC patients from two publicly accessible sources, namely, TCGA-LIHC training cohort and the GSE14520 validation cohort, were fully considered during our investigation.

The endoplasmic reticulum provides a location for the synthesis of intracellular proteins, lipids, and sugars, as well as for the folding and transport of secretory proteins [12–15]. ER can be divided into two types based on the presence or absence of ribosomes attached to its outer membrane, which are known as rough and smooth endoplasmic reticulum [15, 16]. In hepatocytes, smooth endoplasmic reticulum removes lipid-soluble waste products and harmful substances of metabolism [13, 15]. ER stress signalling induced by adverse environmental stimuli, with the feature of abnormal aggregation of misfolded/unfolded proteins, was reported to be linked to modulation of cell fate or the aetiology of many clinical diseases, including cancer [13, 17, 18]. In the present study, we first utilized machine learning to build an ER-associated clinical model with high prognostic accuracy for the outcome assessment of HCC patients. Five ER-related hub genes, KPNA2 (karyopherin *α*2), FMO3 (flavin-containing monooxygenase 3), SPP1 (sphingosine-1-phosphate phosphohydrolase-1), KIF2C (kinesin family member 2C), and LPCAT1 (lysophosphatidylcholine acyltransferase 1), were identified. Furthermore, based on the model, we examined the potential mechanisms in terms of gene expression, Cox regression, nomogram prediction, gene functional enrichment, tumour clustering, and tumour-infiltrating status of immune cells.

## 2. Materials and Methods

### 2.1. Gene Expression Matrix and Clinical Traits

Based on the GSEA (gene set enrichment analysis) online website (http://www.gsea-msigdb.org/gsea/login.jsp), we entered “endoplasmic reticulum” in the “keywords” section, then selected the ER-related items, and finally exported the target genes (*n* = 1975). A “TCGAbiolinks” R package was used to download the gene expression matrix with the type of FPKM-UQ and clinical traits from TCGA-LIHC cohort. Meanwhile, the expression matrix, clinical traits, and annotation files of GSE14520 within the GEO (Gene Expression Omnibus) repository cohort were downloaded by using the “GEOquery” R package. Afterwards, batch correction of the expression matrix within TCGA-LIHC and GSE14520 was carried using a “sva” R package. The clinical characteristics of TCGA-LIHC and GSE14520, such as sex, age, and pathologic stage, are summarized in Supplementary Tables 1-2, respectively.

### 2.2. Differential Gene Screening

We matched clinical characteristics to the expression matrix and then screened for differential genes between normal and HCC tissues with a “limma” R package, setting log2 FC (fold change) to 1 and FDR (false discovery rate) to 0.05. Volcano and MA plots were generated using the “ggplot” R package. Additionally, heatmaps of all differential genes were created using the “pheatmap” R package.

### 2.3. Clinical Prognostic Analysis

After integrating TCGA-LIHC and GSE14520 data matrices with OS and RFS clinical survival status and time, a univariate Cox regression was performed using a “survival” R package with the filter of *p* = 0.05. The corresponding forest plots were created. Furthermore, a multivariate Cox regression analysis with forest plot visualization of the prognostic risk score, sex, age, and pathological stage was performed. Additionally, based on the prognostic signature genes with a series of factors, including age, sex, pathological stage, histologic grade, Eastern Cooperative Oncology Group (ECOG), and vascular tumour cell type, the survival status was estimated using the “surv” R package, and the Kaplan–Meier survival curves were generated using “survminer” R.

### 2.4. Lasso Regression Model Construction

The Venn tool (http://bioinformatics.psb.ugent.be/webtools/Venn/) was used to determine the gene intersections from the univariate Cox regressions and differential expression analyses. TCGA-LIHC was adopted as a training dataset. Based on the intersecting genes, a Lasso regression model was constructed using the “glmnet” R package, and the model was visualized to determine the associated gene coefficients and risk scores. Using the gene coefficients multiplied by the expression level of each hub gene, we calculated the risk scores [8, 10, 19]. The median value of the risk score was used to differentiate high- and low-risk groups. The OS and RFS risk values for the GSE14520 validation group were then computed by the model established by TCGA-LIHC. We then plotted the survival curves for the high- and low-risk groups using the “survivor” and “survminer” R packages. Additionally, the survival status and the heatmap containing the risk score, gene expression, and related clinical features were plotted using the “pheatmap” R package. An ROC analysis of the prognostic risk score, sex, age, and stage was performed using the survivalROC R package, and an AUC (area under the ROC curve) value was calculated.

### 2.5. Nomogram Prediction Model

First, targeting TCGA-LIHC training set, we conducted a modelling analysis using the “regplot” R package. A nomogram was plotted. The “ggstatsplot” R package was utilized to plot the calibration curves for survival rates of 1-, 3-, and 5-year-old HCC patients. Based on the “survIDINRI” R package, the NRI (net reclassification improvement) and IDI (integrated discrimination improvement) values were calculated to explore whether the addition of risk score has an impact on the evaluation efficiency of our model. Finally, we assessed the clinical effectiveness of TCGA-LIHC-OS, GEO-OS, and GEO-RFS by means of decision curve analysis (DCA) with the “ggDCA” R package.

### 2.6. Correlation between Prognostic Gene Expression and Clinical Traits

We matched the expression matrix and clinical information of the ER-related genes from TCGA-LIHC dataset by using an R language approach. The expression differences of hub genes between normal and HCC tissues were analysed by a wilcox.test () R function. The “pheatmap” R package was utilized to obtain the relevant heatmaps, and the “vioplot” R package was used to draw the violin plots. The expression correlations between genes were then analysed and plotted using the “corrplot” and “psych” R packages. Based on the expression data of the target gene set between HCC and paracancerous tissues, we performed a wilcox.test for the paired samples using the “ggpubr” R package. The results were visualized by the ggdotchart () R package. In addition, the protein expression differences of these signatures between HCC and normal tissues were analysed by immunohistochemical images within the HPA (Human Protein Atlas) database.

Furthermore, we analysed the expression differences of these signatures among the different groups of clinical traits, including pathological grade, age, sex, pathological T/N/M, histologic grade, ECOG, vascular tumour cell type, adjacent hepatic tissue inflammation, and tumour status. The statistical correlations between the high/low risk and the continuous variable indices of clinical traits, including height, weight, BMI, creatinine, fetoprotein, albumin, platelets, and prothrombin time, were also analysed. The kruskal.test () R function was applied for the statistical analysis of more than two groups, and the wilcox.test () R function was applied for two groups. The data were visualized as a violin plot, box plot, bar plot, or scatter diagram by using the “ggplot2” or “ggpubr” R packages. Finally, based on the survival status and time of TCGA-LIHC-OS, GEO-OS, and GEO-RFS, we analysed the correlations between gene expression and clinical prognosis of signature genes using the R packages “survival” and “survminer.”

### 2.7. Tumour Cluster Analysis

Based on the expression matrix of prognostic genes, HCC patients of TCGA-LIHC and GSE14520 were clustered by a “ConsensusClusterPlus” R package [20]. Principal component analysis (PCA) was performed by a prcomp () function, and the result was visualized by the “ggplot2” R package. We matched the expression matrices of different tumour clusters and the clinical traits and then presented the results as a heatmap using the “pheatmap” R package. The “survival” R package and plot () were also utilized for cluster-specific prognostic analysis and data visualization.

### 2.8. Gene Enrichment Analysis

Based on the median value of the risk score, TCGA and GEO samples were divided into high- and low-risk groups, and differentially expressed genes were identified using the “limma” R package. A volcano plot was obtained by the “ggplot2” R package. The top ten differentially expressed gene-related heatmaps were generated by the “pheatmap” R package. Next, we utilized the “VennDiagram” R package for an intersection analysis of positively and negatively related gene sets from both TCGA and GEO. The gene enrichment analyses, including GO (Gene Ontology) and KEGG (Kyoto Encyclopedia of Genes and Genomes), were then conducted by the R packages “clusterProfiler,” “org.Hs.e.g.db,” and “pathview,” and the results were visualized by a barplot (). We further conducted gene set enrichment analysis (GSEA) of high/low risk using the GESA software (version 4), and the results were visualized by the R packages “plyr.” “ggplot2,” “grid,” and “gridExtra.”

### 2.9. Tumour-Infiltrating Immune Cell Analysis

The CIBERSORT algorithm [21] allowed us to assess the infiltration levels of immune cells in groups with high- and low-risk TCGA and calculate the percentages of 22 immune cell populations, such as “T cells CD8,” “NK cells activated,” “monocytes,” “macrophages M0/1/2,” and “dendritic cells activated.” Visualizations of the results were created with the “ggplot2” R package. Using the stat_compare_means () function, the difference between the high- and low-risk groups was analysed for the infiltration percentages of immune cells. Based on the ratios of stromal and immune cells, the “ESTIMATE” package [22] was used to determine tumour purity. The results were broken down into three columns: stromal score, immunoscore, and ESTIMATEScore. Using the “ggpubr” R package, a violin plot was created.

## 3. Results

### 3.1. Analysis Strategy

**Figure 1:**
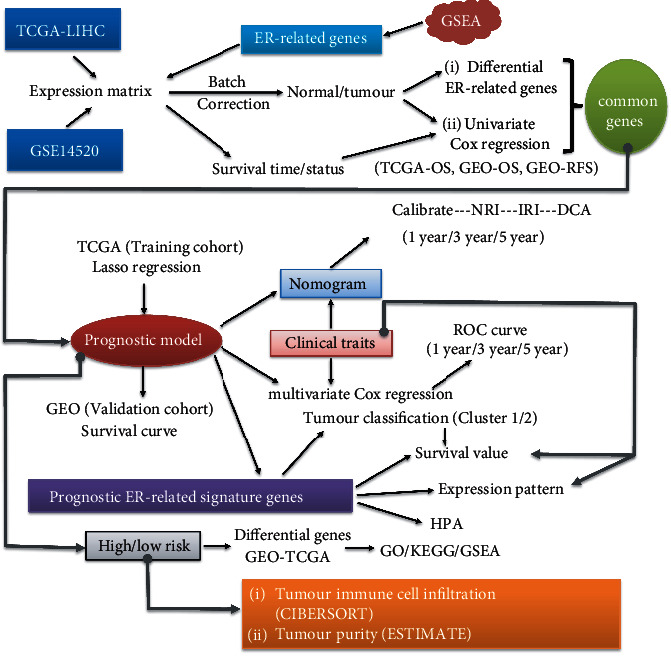
Flow chart of our analysis strategy.

Our study is aimed at developing an ER-related prognostic model based on TCGA and GEO datasets and at exploring the potential molecular mechanisms.  Figure 1 presents our analysis strategy. In brief, we first extracted the expression matrices of TCGA-LIHC and GSE14520 and performed batch correction. Targeting the ER-related genes, the corresponding ER expression matrix was extracted. Then, we conducted differential ER-related gene screening between normal and HCC tissues. In combination with clinical survival status and time, a series of univariate Cox regression analyses (including TCGA-OS, GEO-OS, and GEO-RFS) were also performed to identify prognosis-related ER genes. Based on the common genes of differential expression and univariate Cox regression analyses, we conducted a Lasso regression analysis to build a prognostic model using TCGA-LIHC as a training cohort. With the clinical information, nomogram and related calibration curves at 1-, 3-, and 5-year survival times were plotted. We also calculated the values of the NRI and IRI and conducted decision curve analysis to evaluate the discrimination and clinical effectiveness of ER-related prognostic prediction signatures. We also performed a multivariate Cox regression analysis in combination with the clinical traits. Subsequent validation based on the prognostic model was carried out in a validation GSE14520 cohort, and survival curve results were obtained. In addition, we divided the HCC patients of TCGA-LIHC and GSE14520 into two groups of high/low risk and conducted differential gene analysis to obtain the intersecting genes, followed by GO, KEGG, and GSEA. Targeting the high/low risk groups, tumour-infiltrating immune cell analysis was conducted using a “CIBERSORT” approach, and tumour purity analysis was performed using the “ESTIMATE” approach. Next, the gene sets of the ER-related prognostic model were applied for HCC clustering analysis. The expression patterns and prognostic features of single hub genes, combined with several clinical traits, were analysed.

### 3.2. Lasso Regression Model

**Figure 2:**
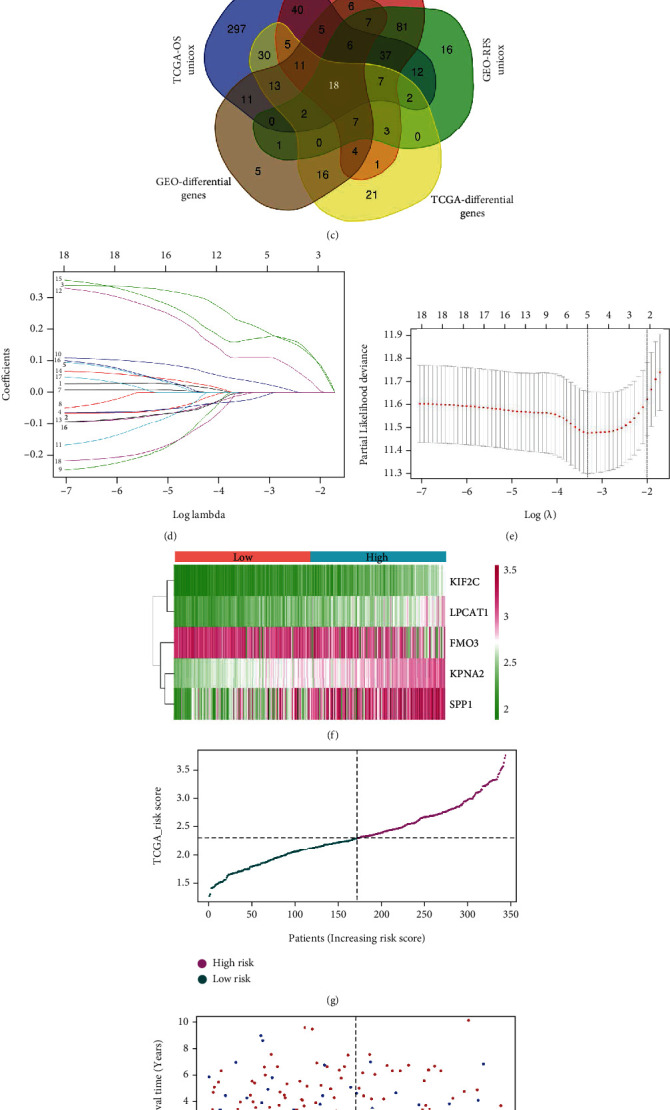
Lasso regression model analysis. Based on the expression matrix of ER-related genes (*n* = 1975) in TCGA-LIHC and GSE14520 cohorts, differential gene analyses between normal and HCC tissues were conducted. (a, b) The MA plots and related heatmaps are presented. (c) Intersection analysis of the ER-related differentially expressed genes and HCC prognosis-related candidate genes of univariate Cox regression analyses was performed. (d, e) Based on a TCGA-LIHC training set, the Lasso regression modelling analysis was performed. We provided the (f) gene expression profile, (g) risk profile, (h) survival status map, and (i) survival curve data of the prognostic model.

Based on the expression matrix of ER-related genes (*n* = 1975) in TCGA-LIHC and GSE14520 cohorts, we conducted differential gene analysis between normal and HCC tissues. As shown in Figure 2(a), compared with normal tissues, we identified 102 ER-related genes highly expressed in the tumour tissues and 38 poorly expressed genes from TCGA-LIHC dataset. In addition, there were a total of 112 ER-related differential genes from the GSE14520 dataset (Figure 2(b)). The related heatmaps are presented in Figures 2(a) and 2(b). We then combined the information of survival status and time to conduct a series of univariate Cox regression analyses to identify a set of HCC prognosis-related candidate genes (Supplementary Figure 1, TCGA-OS, *n* = 496; GEO-OS, *n* = 313; and GEO-RFS, *n* = 199). Subsequently, we performed an intersection analysis of these genes with the ER-related differentially expressed genes to obtain eighteen common genes (Figure 2(c)). Using TCGA-LIHC as a training set, the Lasso regression modelling analysis (Figures 2(d) and 2(e)) was then conducted to obtain five hub genes with correlation coefficients (Coef), namely, KPNA2 (0.213), FMO3 (-0.019), SPP1 (0.0348), KIF2C (0.112), and LPCAT1 (0.172). The risk scores of each sample within the TCGG-LIHC and GSE14520 cohorts were calculated by the following formula: KPNA2 expression × 0.213 − FMO3 expression × 0.019 + SPP1 expression × 0.0348 + KIF2C expression × 0.112 + LPCAT1 expression × 0.172.

We classified the HCC samples into low- and high-risk groups based on the risk scores and then generated heatmaps for the gene expression profiles of the prognostic model of TCAG-LIHC and GSE14520 (Figure 2(f), Supplementary Figure 2(a)), risk profiles (Figure 2(g), Supplementary Figure 2(b)), survival status maps (Figure 2(h), Supplementary Figure 2(c)), survival curves (Figure 2(i), Supplementary Figure 2(d)-2(e)), and heatmaps combining clinical traits (Supplementary Figure 3-4). We observed a higher mortality rate in HCC cases with higher risk values (Figure 2(h), Supplementary Figure 2(c)). After analysing the differences in height, weight, BMI, creatinine, fetoprotein, albumin, platelets, and prothrombin time for TCGA-LIHC cases between the high- and low-risk groups (Supplementary Figure 5(a)-5(h)), we observed a slightly higher level of fetoprotein (Supplementary Figure 5(e), *p* = 0.019) but a slightly lower level of albumin (Supplementary Figure 5(f), *p* = 0.041) in the high-risk group, than in the low-risk group. HCC patients with a high-risk profile had a worse prognosis for survival than those with a low-risk profile (Figure 2(i), *p* = 4.822*e* − 09 for TCGA-OS; Supplementary Figure 2(d), *p* = 4.331*e* − 03 for GEO-OS; Supplementary Figure 2(e), *p* = 4.492*e* − 03 for GEO-RFS).

### 3.3. Expression Patterns of Hub Genes

**Figure 3:**
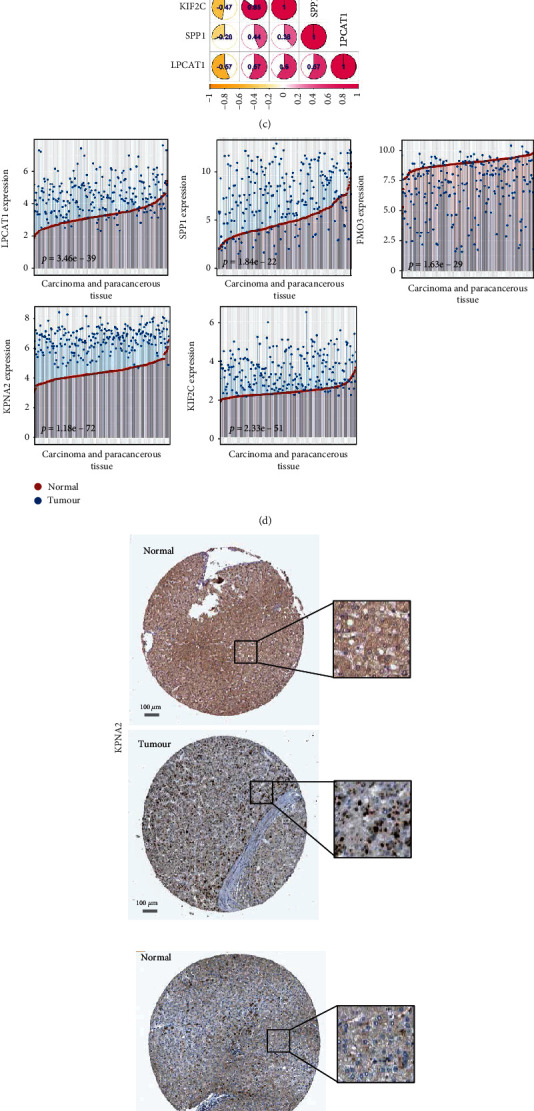
Expression pattern analysis of hub genes. We analysed the expression difference of each hub gene between HCC and normal tissues for TCGA-LIHC cohort. The (a) heatmap, (b) violin plot, and (c) expression correlation plot are shown. We also analysed the expression difference of hub genes between the HCC and paracancerous tissues. (d) The dot plots were provided, and a wilcox.test was conducted. (e) Histochemical results of KPNA2 and LPCAT1 proteins within the HPA database are presented. Bar, 100 *μ*m.

We matched the expression matrix and the corresponding clinical traits for the expression pattern analysis for each hub gene of our model. As shown in Figures 3(a) and 3(b), there were high expression levels of KPNA2, KIF2C, SPP1, and LPCAT1 genes (*p* < 0.001) but a low expression level of FMO3 (*p* < 0.001) in HCC tissues compared with normal tissues within TCGA-LIHC cohort. Further expression correlation analysis (Figure 3(c)) suggested that FMO3 showed negative correlations (*r* < 0, all *p* < 0.001) of gene expression with KPNA2, KIF2C, SPP1, and LPCAT1. In addition, we observed positive correlations among KPNA2, KIF2C, SPP1, and LPCAT1 genes (Figure 3(c), *r* > 0, all *p* < 0.001). Of them, there was the greatest significant difference for the correlation between KPNA2 expression and KIF2C expression (Figure 3(c), *r* = 0.85, *p* = 5.09*e* − 120).

We also investigated the expression features of these five genes in paired HCC and paracancerous tissues from the GSE1520 cohort. As shown in Figure 3(d), there were higher expression levels of LPCAT1 (*p* = 3.46*e* − 39), SPP1 (*p* = 1.84*e* − 22), KPNA2 (*p* = 1.18*e* − 72), and KIF2C (*p* = 2.33*e* − 51) and a lower expression level of FMO3 (*p* = 1.63*e* − 29) in HCC tissues than in the matched paraneoplastic tissues. The immunohistochemical analysis data of the HPA database further indicated higher KPNA2 and LPCAT1 protein staining signals in HCC tissues than in normal tissues (Figure 3(e)). Histochemical data of LPCAT1, SPP1, and FMO3 were temporarily unavailable in the HPA database.

Next, we analysed the statistical correlations between hub gene expression and clinical traits (Supplementary Figure 6-9) within TCGA-LIHC cohort. Briefly, the expression patterns of KPNA2, FMO3, SPP1, KIF2C, and LPCAT1 were statistically correlated with the clinical stage (Supplementary Figure 6(a), *p* < 0.001), especially the pathological T stage (Supplementary Figure 7, *p* < 0.05). A similar result was detected for histologic grade (Supplementary Figure 8(a), *p* < 0.01). Our findings also revealed significant correlations between KPNA2, LPCAT1, KIF2C, and SPP2 expression and three clinical traits, including ECOG (Supplementary Figure 8(b), *p* < 0.01), vascular tumour cell type (Supplementary Figure 9(a), *p* < 0.05), and tumour status (Supplementary Figure 9(c), *p* < 0.05). Additionally, there were correlations between SPP1 expression and age (Supplementary Figure 6(b), *p* < 0.05), FMO3 expression and sex (Supplementary Figure 6(c), *p* < 0.001), and SPP1 expression and adjacent hepatic tissue inflammation (Supplementary Figure 9(b), *p* < 0.05). For the GSE14520 cohort, we observed statistical correlations of stage with the expression levels of KPNA2, FMO3, SPP1, and LPCAT1 (Supplementary Figure 6(d), *p* < 0.01). In addition, the age factor was correlated with the expression of KIF2C and LPCAT1 (Supplementary Figure 6(e), *p* < 0.01), while the sex factor was linked to the expression of SPP1 (Supplementary Figure 6(f), *p* < 0.01).

### 3.4. Prognostic Analysis of Hub Genes

**Figure 4:**
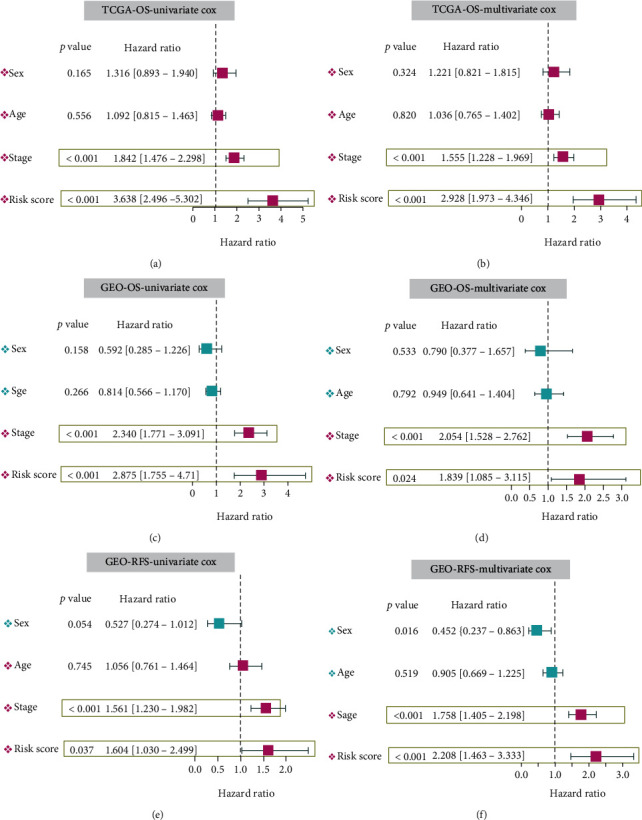
Univariate/multivariate Cox regression analyses of risk score. Targeting the factors of prognostic risk score, sex, age, and stage, we conducted univariate/multivariate Cox regression analyses for the prognosis of OS or RFS. (a, b) TCGA-LIHC OS; (c, d) GSE14520 OS; (e, f) GSE14520 RFS.

We also conducted prognostic analyses of TCGA-OS, GEO-OS, and GEO-RFS for each hub gene of our model. As presented in Supplementary Figure 10, HCC patients with high expression levels of KPNA2, KIF2C, SPP1, and LPCAT1 and low expression of FMO3 exhibited a poor clinical prognosis of OS and RFS. Statistically significant differences were observed in all groups (Supplementary Figure 10(a)-10(c), *p* < 0.05), except for KPNA2 in GSE14520 (Supplementary Figure 10(c), *p* = 1.496*e* − 01). When we combined the risk score and the factors of age (Supplementary Figure 11(a)-11(b)), sex (Supplementary Figure 11(c)-11(d)), pathological stage (Supplementary Figure 12(a)), histologic grade (Supplementary Figure 12(b)), ECOG (Supplementary Figure 12(c)), and vascular tumour cell type (Supplementary Figure 12(d)), we still obtained positive conclusions (all *p* < 0.05). Based on the risk score of each case and the corresponding clinical traits (sex, age, and stage), univariate/multivariate Cox regression analyses were performed as well. The results in Figures 4(a)–4(f) show that both the stage and risk score were statistically correlated with the clinical prognosis of HCC patients. The higher the stage level and risk score, the worse the prognosis of OS and RFS in the HCC cases of both TCGA-LIHC and GSE14520 cohorts (all HR > 1, *p* < 0.05). Subsequent ROC results indicated that the stage and risk score factors had good performance in predicting 1-, 3-, and 5-year survival rates (Supplementary Figure 13(a)-13(c), AUC > 0.6).

### 3.5. Tumour Clustering Analysis

**Figure 5:**
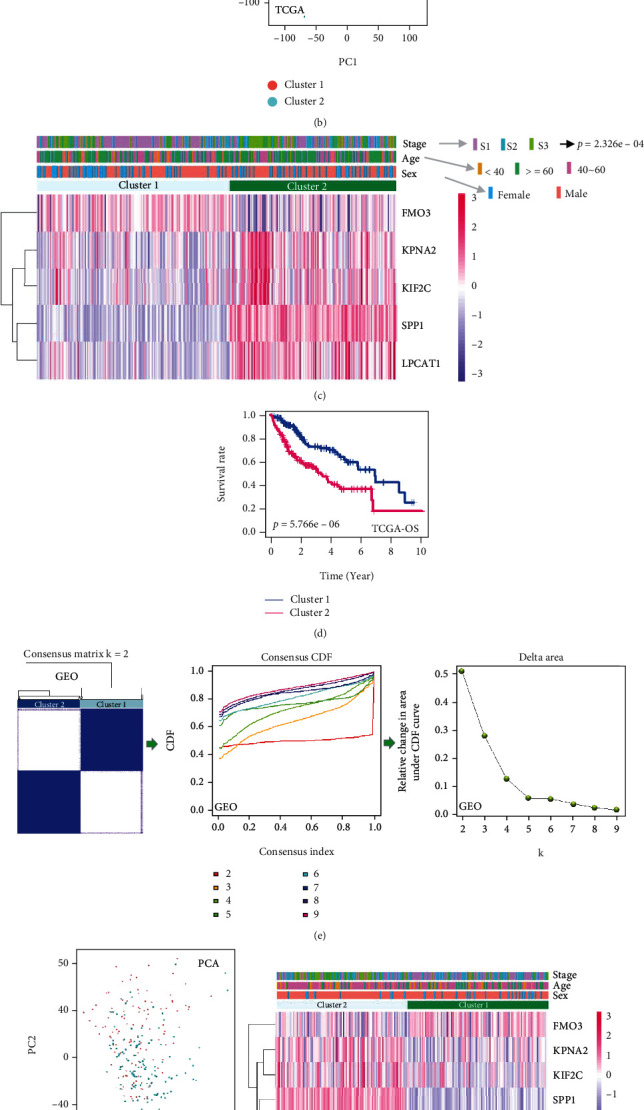
Tumour clustering analysis of hub genes. (a) Based on the expression matrix of prognostic genes, HCC patients of TCGA-LIHC were clustered. (b) PCA was conducted to evaluate the classification effect on tumour clusters. (c) The heatmap of hub gene expression and clinical traits and (d) the cluster-specific prognostic curve of OS for TCGA-LIHC cohort are presented. (e–h) We also performed a similar tumour clustering analysis for the HCC patients of GSE14520.

TCGA-LIHC samples were analysed using hub genes to identify two tumour clusters (Figure 5(a)). Subsequently, we separated these two clusters of tumours using a PCA approach (Figure 5(b)). Combined with clinical characteristics, we presented heatmaps of hub gene expression for different tumour clusters and observed a significant difference between the two tumour clusters and stages (Figure 5(c), *p* = 2.326*e* − 04). Compared to Cluster 1, the Cluster 2 group showed high expression levels of FPNA2, KIF2C, SPP1, and LPCAT1 and a low expression level of FMO3 (Figure 5(c)). In addition, HCC patients in the Cluster 2 group had a poorer prognosis of OS than those in the Cluster 1 group (Figure 5(d), *p* = 5.766*e* − 06). We also conducted tumour clustering analysis of the GSE14520 cohort and obtained two similar tumour clusters (Figure 5(e)), which could also be classified by a PCA approach (Figure 5(f)). The two clusters were associated with the clinical stage factor (Figure 5(g), *p* = 2.74*e* − 05). Compared with Cluster 1, HCC patients in Cluster 2 had a worse prognosis for OS and RFS (Figure 5(h), *p* = 0.02 for OS; *p* = 1.575*e* − 04 for RFS).

### 3.6. Nomogram and Related Assessments

**Figure 6:**
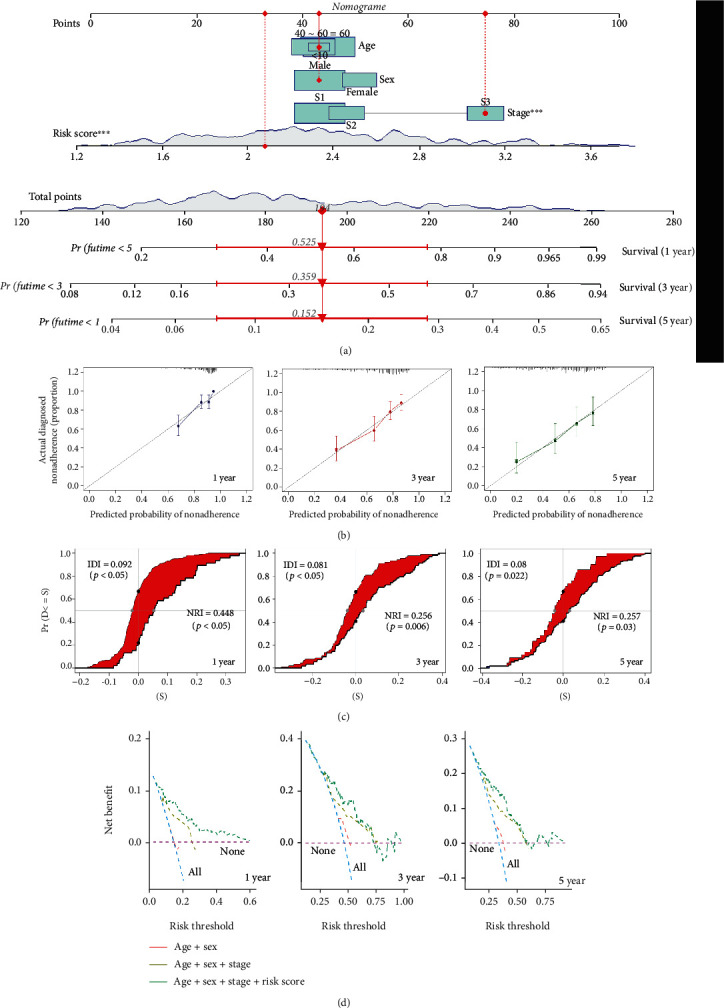
Nomogram and related assessment analyses. (a) A nomogram was plotted to predict the 1-, 3-, and 5-year survival rates of a given HCC patient within TCGA-LIHC cohort, ^∗∗∗^*p* < 0.001. We also provided the data for (b) the calibration plot curve, (c) assessment of NRI/IDI, and (d) DCA for the 1-, 3-, and 5-year survival times, respectively.

First, using TCGA-LIHC training set, we developed a nomogram for predicting the 1-, 3-, and 5-year survival rates of a given case of HCC according to the combined factors of risk score, stage, age, and sex. As shown in Figure 6(a), combining the risk score, stage, age, and sex information of the selected patients, we were able to predict the survival rates for 1, 3, and 5 years, which were 0.525, 0.359, and 0.152, respectively. A high degree of overlap is evident in the calibration plot curve of Figure 6(b). The values of NRI and IDI indicated that the prediction effects of the model for 1-, 3-, and 5-year survival time improved after adding the risk score factor (Figure 6(c), all IDI > 0, NRI > 0, and *p* < 0.05). Our DCA data of TCGA-LIHC (Figure 6(d)) and GSE14520 (Supplementary Figures 14(a)-14(b)) further exhibited better clinical validity in the “age + sex + stage + risk score” group than in the other groups.

### 3.7. Prognostic Risk-Related Differential Gene Analysis

**Figure 7:**
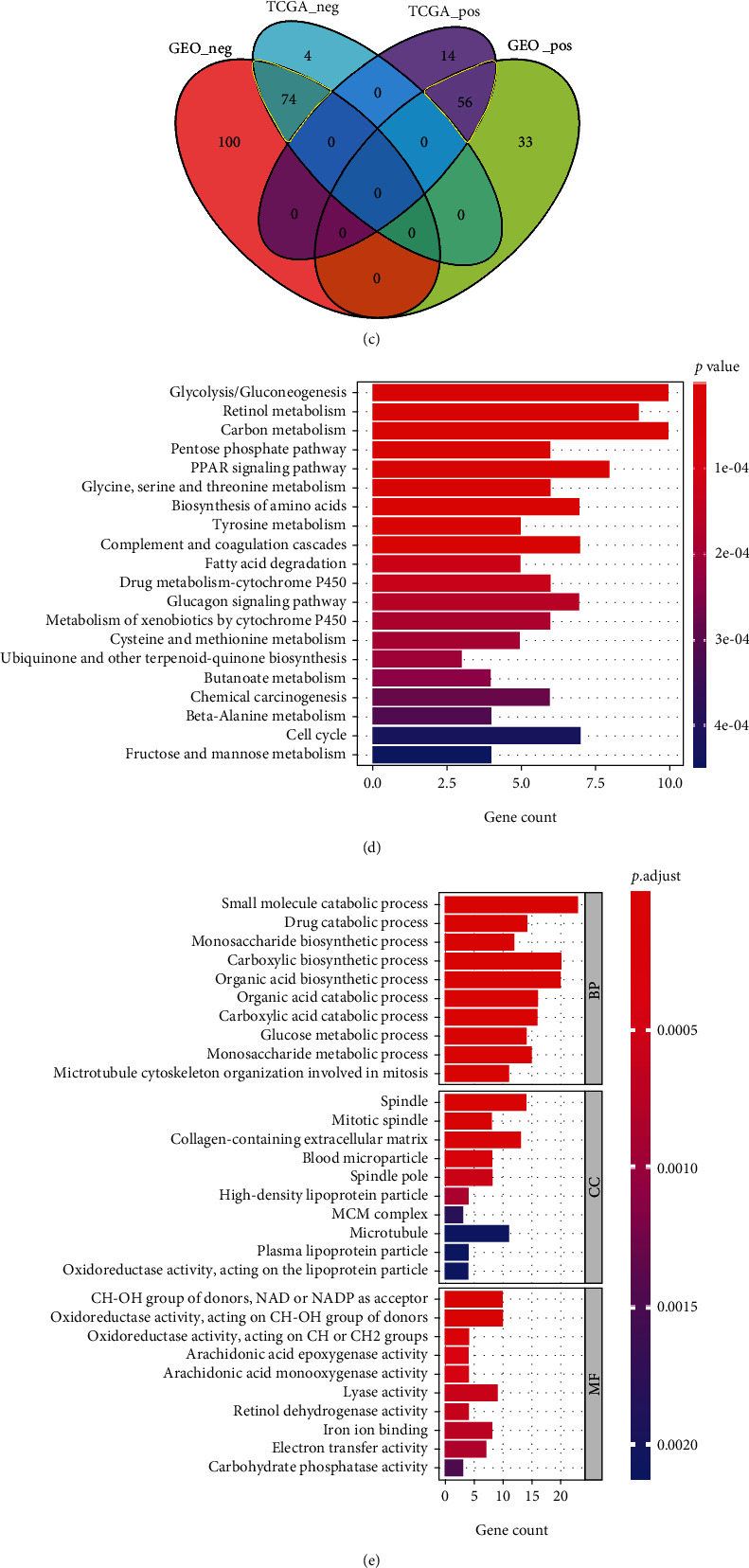
Prognostic risk-related differential gene analysis. We conducted differential gene identification between the high- and low-risk groups and visualized the data as a volcano plot and a heatmap of the top ten differential genes in both (a) TCGA-LIHC and (b) GSE14250. (c) Then, an intersection analysis of positively and negatively related gene sets of both TCGA and GSE14250 was conducted to identify the common genes. Gene enrichment analyses of (d) KEGG, (e) GO, and (f) GSEA were performed.

We used the “limma” R package to identify differentially expressed genes using high/low-risk grouping in TCGA-LIHC and GSE14520 (Figures 7(a) and 7(b)). Based on an intersection analysis, 74 genes negatively associated with high risk and 56 positively associated genes were identified (Figure 7(c)). Using these differentially expressed genes, we carried out the GO and KEGG enrichment analyses (Figures 7(d) and 7(e)). These genes were associated with biological enzyme activity, biological metabolic processes, or the cell cycle, e.g., “small molecule catabolic process,” “CH-OH group of donors, NAD or NADP as acceptor,” “glycolysis/gluconeogenesis,” “carbon metabolism,” “microtubule cytoskeleton organization involved in mitosis,” “spindle,” or “mitotic spindle.” Additionally, GSEA results revealed a series of genes related to G1/S, G2/M checkpoints, and E2F targets (Figure 7(f)).

### 3.8. Tumour-Infiltrating Immune Cell Analysis

**Figure 8:**
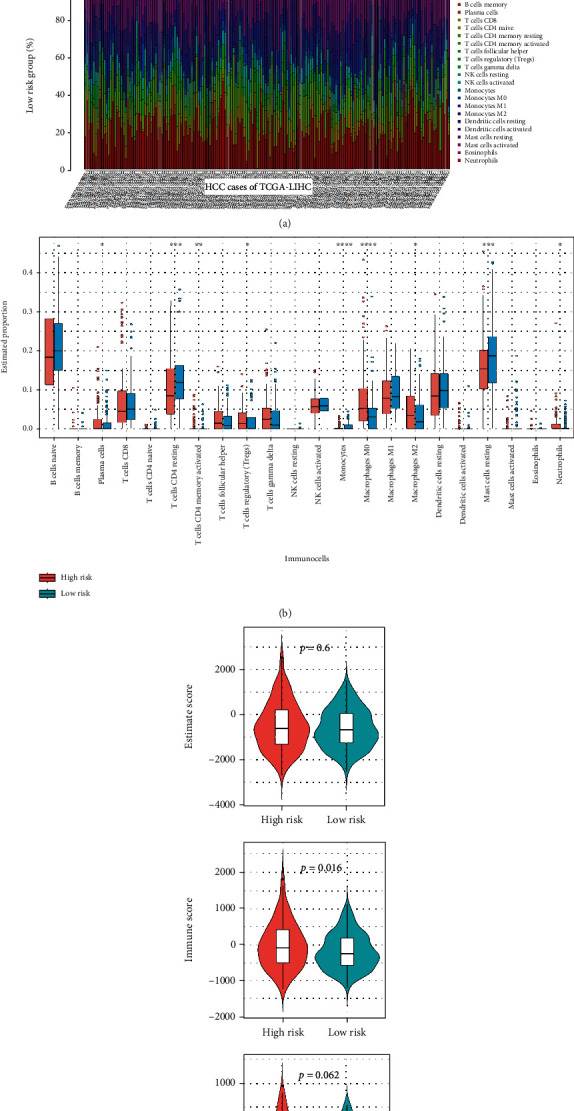
Tumour-infiltrating immune cell analysis. Based on a “CIBERSORT” approach, the tumour infiltration levels of 22 immune cell populations in the high- and low-risk groups were calculated. The results were visualized as (a) a stacking percentage histogram and (b) a boxplot. We also evaluated the tumour purity using an “ESTIMATE” approach. (c) Violin plots of the stromal score, immune score, and ESTIMATEScore are shown.

Finally, we utilized a “CIBERSORT” algorithm to obtain the proportions of 22 immune cell populations of each HCC patient, such as “T cells CD8,” “NK cells activated,” “monocytes,” “macrophages M0/1/2,” and “dendritic cells activated,” in the high- and low-risk groups of TCGA-LIHC cohort (Figure 8(a)). Compared with the low-risk group, the proportions of “T cells CD4 resting” and “mast cells resting” were relatively low in the high-risk group (Figure 8(b), *p* < 0.001), while “plasma cells,” “regulatory T cells (Tregs),” “macrophages M0,” “macrophages M2,” and “neutrophils” were relatively high (Figure 8(b), *p* < 0.05). Additionally, we utilized the “Estimate” algorithm to estimate the tumour purity. Even though we did not observe significant differences in the overall tumour purity (Figure 8(c), ESTIMATEScore, *p* = 0.6) and stromal cell (*p* = 0.062) ratios between the high- and low-risk groups, there was a relatively high percentage of immune cells in the high-risk group compared with the low-risk group (*p* = 0.016). Collectively, these results indicated a correlation between ER prognostic model gene-related high/low risk and tumour infiltration of immune cells.

## 4. Discussion

Growing evidence indicates the functional links between endoplasmic reticulum-related events and the occurrence, development, and even clinical immunotherapy of cancers [23–26]. In 2021, Liu et al. published an ER stress- (ERS-) related HCC prognostic model based on TCGA-LIHC and ICGC (International Cancer Genome Consortium) datasets [27]. Five genetic variables, including HDGF, EIF2S1, SRPRB, PPP2R5B, and DDX11, were narrowed down from 88 ERS genes by means of a univariate/multivariate Cox regression approach [27]. In the current study, we focused on the ER rather than the ERS. For our ER-related prognostic model, we enrolled the different datasets from the HCC cohorts of TCGA-LIHC and GSE14520 and mainly utilized the modelling strategy approach of Lasso regression and the model effectiveness assessment methods, including ROC, calibration curves, NRI, IDI, and DCA. Finally, five prognostic gene signatures, SPP1, KIF2C, LPCAT1, KPNA2, and FMO3, were identified from a total of 1975 ER-related genes, and a high assessment power was detected for the combined panel. Furthermore, we first identified two tumour clusters by consensus clustering that were closely correlated with the survival prognosis of HCC patients and provided evidence regarding the correlation between our ER-related models and a set of events, including biological enzyme activity, biological metabolic processes, cell cycle, and tumour infiltration of immune cells.

Several publications have reported the relationships between these five ER-related genes (SPP1, KIF2C, LPCAT1, KPNA2, and FMO3) and cancers. SPP1, located in the ER, is an enzyme responsible for the dephosphorylation of intracellular S1P (sphingosine-1-phosphate) [28, 29]. SPP1 is related to the upregulation of autophagy and apoptosis upon ER stress [28–30]. High expression of SPP1 could promote the proliferation of HCC cells [31]. KIF2C increased the proliferation or migration ability of HCC cell lines and aggravated HCC progression, indicating a potential therapeutic biomarker for clinical HCC treatment [32–34]. LPCAT1, as an ER-resident protein, could participate in the conversion of lysophosphatidylcholine into phosphatidylcholine [35]. Highly expressed LPCAT1 was observed in several types of cancer tissues compared with normal tissues [35–37]. It was reported that LPCAT1 affected the phospholipid composition of HCC cells and modulated the progression of HCC [36, 37]. KPNA2 is involved in the nucleoplasm shuttle process of various oncoproteins, and highly expressed KPNA2 is linked to poor prognosis in patients with some kinds of cancer [38–40]. FMO3 was implicated in the modulation of cholesterol metabolism [41] and radical production in the endoplasmic reticulum [42]. It was reported that FMO3 could participate in the regulation of glucose metabolism in the liver by reducing lipid-induced ER stress [43]. Herein, we observed high expression levels of KPNA2, KIF2C, SPP1, and LPCAT1 genes but low expression levels of FMO3 in HCC tissues. The KPNA2, LPCAT1, KIF2C, and SPP2 genes were statistically correlated with pathological stage, histologic grade, ECOG, vascular tumour cell type, and tumour status.

In our study, we evaluated the expression patterns and potential prognostic significance of five hub genes in HCC patients within TCGA-LIHC and GSE14520 cohorts. FMO3 presented the opposite result compared to other proteins. Briefly, HCC patients with high KPNA2, KIF2C, SPP1, and LPCAT1 expression but low FMO3 expression showed a poor survival prognosis. By combining the expression features of the five genes, we observed an improved prognostic value for OS or RFS for the 1-, 3-, and 5-year survival periods. Positive conclusions were obtained when adding the factors age, sex, and pathological stage. In addition, we obtained two clusters by consensus clustering of ER-related genes for HCC patients in TCGA-LIHC and GSE14520 cohorts. HCC patients in Cluster 2 with high KPNA2, KIF2C, SPP1, and LPCAT1 expression and low FMO3 expression showed a worse survival prognosis than HCC patients in Cluster 1.

To stratify HCC risk, a five-gene-based prognostic signature model was established by TCGA-LIHC training dataset and verified by the GSE14520 testing dataset. Good clinical prognostic competence and survival prediction accuracy were observed for our model. Furthermore, the model-based nomogram is of potential clinical application significance to predict the survival prognosis of HCC patients. In addition, we performed a series of functional enrichments based on the differentially expressed genes between the high- and low-risk groups. Due to the links of all five hub genes for risk classification and the endoplasmic reticulum, we enriched the terms of biological enzyme activity or metabolic processes as expected. KPNA2 may have an impact on the biological behaviours of HCC by regulating DNA replication and the cell cycle [40]. We observed several enrichments in cell cycle markers, including the G1/S and G2/M checkpoints.

Growing evidence supports the point that the tumour immune microenvironment is essential for the pathological process of HCC [44–46]. A set of immune cell infiltration-associated genes reportedly showed a prognostic effect for HCC patients [33, 47]. KPNA2 expression was also positively correlated with the immune infiltration levels of B cells in HCC tissues [48]. In the present study, we investigated the microenvironmental infiltration feature for the ER model-based high/low risk groups of HCC cases through two algorithms, namely, “ESTIMATE” and “CIBERSORT.” The immune cells and stroma are the two main kinds of nontumor components within the tumour microenvironment [22]. Our finding of “ESTIMATE” indicated a relatively high infiltration proportion of immune cells in the high-risk group compared to the low-risk group. Specifically, we observed a relatively high fraction of “plasma cells” in the high-risk group compared with the low-risk group. A single-cell RNA sequencing study reported that there was a higher infiltration level of “plasma cells” in tumours of HCC with cirrhosis compared with the normal controls [49]. There was a poor survival prognosis for TCGA HCC cases with a high infiltration level of plasma cells [49]. Similarly, the HCC cases in the high-risk group exhibited a worse OS or RFS prognosis than those in the low-risk group.

Furthermore, we detected higher infiltration levels of “macrophages M2/M0” and “regulatory T cells (Tregs)” in the high-risk group than in the low-risk group. Macrophages with alternatively activated (M2) phenotypes are implicated in promoting HCC tumorigenesis [50]. In addition to “macrophages M2,” HCC patients in the cluster with enriched “macrophages M0” also exhibited a poor clinical prognosis [51]. A previous meta-analysis reported the relationship between a high infiltration status of Tregs and shorter survival time for HCC patients [52]. These findings may partly explain the poorer OS prognosis status of HCC cases in the high-risk group. Our proposed ER-related prognostic signature may be involved in the immunotherapeutic strategies of HCC.

Even though our ER-related risk model showed a high prediction performance for HCC prognosis, there were still some limitations. For instance, due to the lack of enough clinical drug treatment of HCC patients, especially for the GSE14520 cohort, we cannot adjust the effects of these clinical management factors. Our five signature-based risk model was developed by the retrospective investigation of two available sources, which still merits further validation of multicentre external datasets. Additionally, more experimental evidence is needed to confirm the potential functional links between our model and biological events, especially tumour immunity.

## 5. Conclusion

Taken together, we integrated the expression matrix of ER-related genes and clinical traits within both TCGA-LIHC and GSE14520 cohorts to develop and validate a novel ER-associated clinical prognostic model of HCC patients, which contains the combined panel of five hub genes (KPNA2, FMO3, SPP1, KIF2C, and LPCAT1). Additionally, we plotted a nomogram with a better clinical survival prediction performance, which may serve as a prognostic predictor to help clinicians assess the clinical survival outcomes of HCC patients.

## Data Availability

The data and materials can be obtained by contacting the corresponding author.

## Supplementary Materials

Supplementary 1 Supplementary Figure 1: univariate Cox regression analyses of TCGA-LIHC and GSE14520. We conducted univariate Cox regression analyses to identify a set of HCC prognosis-related candidate genes for TCGA-LIHC OS (a), GSE14520 OS (b), and RFS (c). Supplementary Figure 2: validation analysis of the Lasso regression model. Based on the risk scores of the Lasso regression model, we divided the HCC patients of GSE14520 into high- and low-risk groups. The corresponding heatmaps (a), risk profiles (b), survival status maps (c), survival curves of OS (d), and RFS (e) are shown. Supplementary Figure 3: heatmap for the hub gene expression and clinical traits of HCC patients within TCGA-LIHC cohort. Supplementary Figure 4: heatmap for the hub gene expression and clinical traits of HCC patients within the GSE14520 cohort. Supplementary Figure 5: correlations between the continuous variable index of clinical traits and high/low risk. The differences in the continuous variable index for TCGA cohorts between the high and low groups were analysed by the wilcox.test: height (a), weight (b), BMI (c), creatinine (d), fetoprotein (e), albumin (f), platelet count (g), and prothrombin time (h). Supplementary Figure 6: correlation analysis between hub gene expression and the factors of pathological stage and age or sex. We combined the expression matrix and clinical information of five hub genes from TCGA-LIHC and GSE14520 cohorts and analysed the expression characteristics for the different pathological stages (a, d) and age (b, e), or sex (c, f), using kruskal.test or wilcox.test. ^∗^*p* < 0.05, ^∗∗^*p* < 0.01, ^∗∗∗^*p* < 0.001. Supplementary Figure 7: correlation analysis between hub gene expression and pathological T/N/M. The expression differences in the five hub genes in the different pathological T/N/M groups were analysed by the kruskal.test, followed by the wilcox.test for TCGA cohort. (a) FMO3; (b) KIF2C; (c) KPNA2; (d) LPCAT1; (e) SPP1. Supplementary Figure 8: correlation analysis between hub gene expression and factors of histologic grade and ECOG. The expression differences of five hub genes in the different histologic grade (a) or ECOG (b) groups were analysed by the kruskal.test followed by the wilcox.test for TCGA cohort. Supplementary Figure 9: correlation analysis between hub gene expression and three factors. The expression differences of five hub genes in the different groups of vascular tumour cell type (a), adjacent hepatic tissue inflammation (b), and tumour status (c) were analysed by the kruskal.test and wilcox.test for TCGA cohort. ^∗^*p* < 0.05, ^∗∗^*p* < 0.01, ^∗∗∗^*p* < 0.001. Supplementary Figure 10: prognostic analyses of hub genes. The survival status of LPCAT1, KPNA2, SPP1, KIF2C, and FMO3 was analysed, and the corresponding Kaplan–Meier survival curves were plotted for TCGA-LICH OS (a), GSE14520 OS (b), and GSE14520 RFS (c). Supplementary Figure 11: prognostic analysis of high/low risk combined with age and sex. The survival status of high/low risk combined with the factors of age and sex was estimated, and the corresponding Kaplan–Meier survival curves were plotted. TCGA-LICH age (a), GSE14520 age (b), TCGA-LICH sex (c), and GSE14520 sex (d). Supplementary Figure 12: prognostic analysis of high/low risk combined with the other four factors. The survival status of high/low risk combined with the factors of pathological stage (a), histologic grade (b), ECOG (c), and vascular tumour cell type (d) were estimated, and the corresponding Kaplan–Meier survival curves were plotted for TCGA-LICH. Supplementary Figure 13: ROC analysis of the risk score. Targeting the factors of prognostic risk score, sex, age, and stage, we conducted ROC analyses for the prognosis of TCGA-LICH OS (a), GSE14520 OS (b), and GSE14520 RFS (c) for the 1-, 3-, and 5-year survival times. Supplementary Figure 14: decision curve analyses for GEO14520. We conducted DCA of GSE14520 OS (a) and RFS (b) using the three groups of “age + sex,” “age + sex + stage,” and “age + sex + stage + risk score” for the 1-, 3-, and 5-year survival times, respectively.

Supplementary 2 Table S1: clinical characteristics of HCC cases for TCGA-LIHC. Table S2: clinical characteristics of HCC cases for GSE14520.
