# Supplementary material for: Identifying a Novel Endoplasmic Reticulum-Related Prognostic Model for Hepatocellular Carcinomas
Source: Oxid Med Cell Longev. 2022 Jul 22;2022:8248355. doi: 10.1155/2022/8248355 (PMC9338738; doi:10.1155/2022/8248355)
Supplement: Supplementary 1 — Supplementary Figure 1: univariate Cox regression analyses of TCGA-LIHC and GSE14520. We conducted univariate Cox regression analyses to identify a set of HCC prognosis-related candidate genes for TCGA-LIHC OS (a), GSE14520 OS (b), and RFS (c). Supplementary Figure 2: validation analysis of the Lasso regression model. Based on the risk scores of the Lasso regression model, we divided the HCC patients of GSE14520 into high- and low-risk groups. The corresponding heatmaps (a), risk profiles (b), survival status maps (c), survival curves of OS (d), and RFS (e) are shown. Supplementary Figure 3: heatmap for the hub gene expression and clinical traits of HCC patients within TCGA-LIHC cohort. Supplementary Figure 4: heatmap for the hub gene expression and clinical traits of HCC patients within the GSE14520 cohort. Supplementary Figure 5: correlations between the continuous variable index of clinical traits and high/low risk. The differences in the continuous variable index for TCGA cohorts between the high and low groups were analysed by the wilcox.test: height (a), weight (b), BMI (c), creatinine (d), fetoprotein (e), albumin (f), platelet count (g), and prothrombin time (h). Supplementary Figure 6: correlation analysis between hub gene expression and the factors of pathological stage and age or sex. We combined the expression matrix and clinical information of five hub genes from TCGA-LIHC and GSE14520 cohorts and analysed the expression characteristics for the different pathological stages (a, d) and age (b, e), or sex (c, f), using kruskal.test or wilcox.test. ∗p < 0.05, ∗∗p < 0.01, ∗∗∗p < 0.001. Supplementary Figure 7: correlation analysis between hub gene expression and pathological T/N/M. The expression differences in the five hub genes in the different pathological T/N/M groups were analysed by the kruskal.test, followed by the wilcox.test for TCGA cohort. (a) FMO3; (b) KIF2C; (c) KPNA2; (d) LPCAT1; (e) SPP1. Supplementary Figure 8: correlation analysis between hu [file 8248355.f1.zip › Figure S11.pptx]

## Slide 1
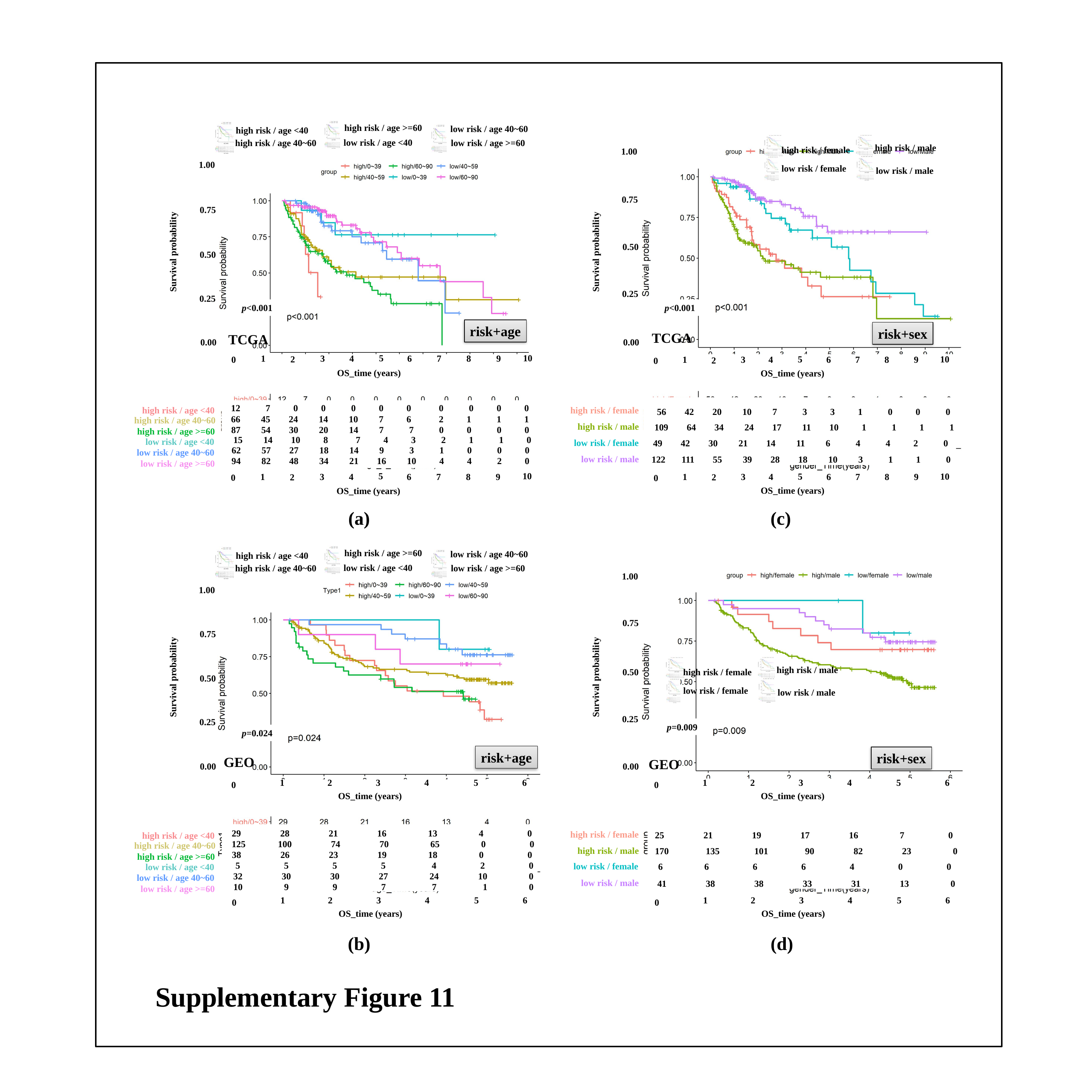

high risk / age >=60
low risk / age 40~60
high risk / age <40
low risk / age <40
low risk / age >=60
high risk / age 40~60
1.00
0.75
Survival probability
0.50
0.25
p<0.001
0.00
5
10
6
7
8
9
1
3
4
2
0
OS_time (years)
12 7 0 0 0 0 0 0 0 0 0
66 45 24 14 10 7 6 2 1 1 1
87 54 30 20 14 7 7 0 0 0 0
15 14 10 8 7 4 3 2 1 1 0
62 57 27 18 14 9 3 1 0 0 0
94 82 48 34 21 16 10 4 4 2 0
high risk / age <40
high risk / age 40~60
high risk / age >=60
low risk / age <40
low risk / age 40~60
low risk / age >=60
5
10
6
7
8
9
1
3
4
2
0
OS_time (years)
(a)
high risk / age >=60
low risk / age 40~60
high risk / age <40
low risk / age <40
low risk / age >=60
high risk / age 40~60
1.00
0.75
Survival probability
0.50
0.25
p=0.024
0.00
2
3
4
5
6
1
0
OS_time (years)
 29 28 21 16 13 4 0
high risk / age <40
high risk / age 40~60
high risk / age >=60
low risk / age <40
low risk / age 40~60
low risk / age >=60
125 100 74 70 65 0 0
38 26 23 19 18 0 0
 5 5 5 5 4 2 0
 32 30 30 27 24 10 0
10 9 9 7 7 1 0
2
3
4
5
6
1
0
OS_time (years)
(b)
high risk / male
high risk / female
low risk / male
low risk / female
1.00
0.75
0.50
Survival probability
0.25
p<0.001
0.00
5
10
6
7
8
9
1
3
4
2
0
OS_time (years)
high risk / female
56 42 20 10 7 3 3 1 0 0 0
high risk / male
109 64 34 24 17 11 10 1 1 1 1
low risk / female
49 42 30 21 14 11 6 4 4 2 0
low risk / male
122 111 55 39 28 18 10 3 1 1 0
5
10
6
7
8
9
1
3
4
2
0
OS_time (years)
(c)
1.00
0.75
high risk / male
high risk / female
low risk / male
low risk / female
0.50
Survival probability
0.25
p=0.009
0.00
2
3
4
5
6
1
0
OS_time (years)
high risk / female
high risk / male
low risk / female
low risk / male
25 21 19 17 16 7 0
170 135 101 90 82 23 0
2
3
4
5
6
1
0
OS_time (years)
 6 6 6 6 4 0 0
 41 38 38 33 31 13 0
(d)
Supplementary Figure 11
risk+age
risk+sex
risk+age
risk+sex
TCGA
TCGA
GEO
GEO
