# Supplementary material for: Identifying a Novel Endoplasmic Reticulum-Related Prognostic Model for Hepatocellular Carcinomas
Source: Oxid Med Cell Longev. 2022 Jul 22;2022:8248355. doi: 10.1155/2022/8248355 (PMC9338738; doi:10.1155/2022/8248355)
Supplement: Supplementary 1 — Supplementary Figure 1: univariate Cox regression analyses of TCGA-LIHC and GSE14520. We conducted univariate Cox regression analyses to identify a set of HCC prognosis-related candidate genes for TCGA-LIHC OS (a), GSE14520 OS (b), and RFS (c). Supplementary Figure 2: validation analysis of the Lasso regression model. Based on the risk scores of the Lasso regression model, we divided the HCC patients of GSE14520 into high- and low-risk groups. The corresponding heatmaps (a), risk profiles (b), survival status maps (c), survival curves of OS (d), and RFS (e) are shown. Supplementary Figure 3: heatmap for the hub gene expression and clinical traits of HCC patients within TCGA-LIHC cohort. Supplementary Figure 4: heatmap for the hub gene expression and clinical traits of HCC patients within the GSE14520 cohort. Supplementary Figure 5: correlations between the continuous variable index of clinical traits and high/low risk. The differences in the continuous variable index for TCGA cohorts between the high and low groups were analysed by the wilcox.test: height (a), weight (b), BMI (c), creatinine (d), fetoprotein (e), albumin (f), platelet count (g), and prothrombin time (h). Supplementary Figure 6: correlation analysis between hub gene expression and the factors of pathological stage and age or sex. We combined the expression matrix and clinical information of five hub genes from TCGA-LIHC and GSE14520 cohorts and analysed the expression characteristics for the different pathological stages (a, d) and age (b, e), or sex (c, f), using kruskal.test or wilcox.test. ∗p < 0.05, ∗∗p < 0.01, ∗∗∗p < 0.001. Supplementary Figure 7: correlation analysis between hub gene expression and pathological T/N/M. The expression differences in the five hub genes in the different pathological T/N/M groups were analysed by the kruskal.test, followed by the wilcox.test for TCGA cohort. (a) FMO3; (b) KIF2C; (c) KPNA2; (d) LPCAT1; (e) SPP1. Supplementary Figure 8: correlation analysis between hu [file 8248355.f1.zip › Figure S12.pptx]

## Slide 1
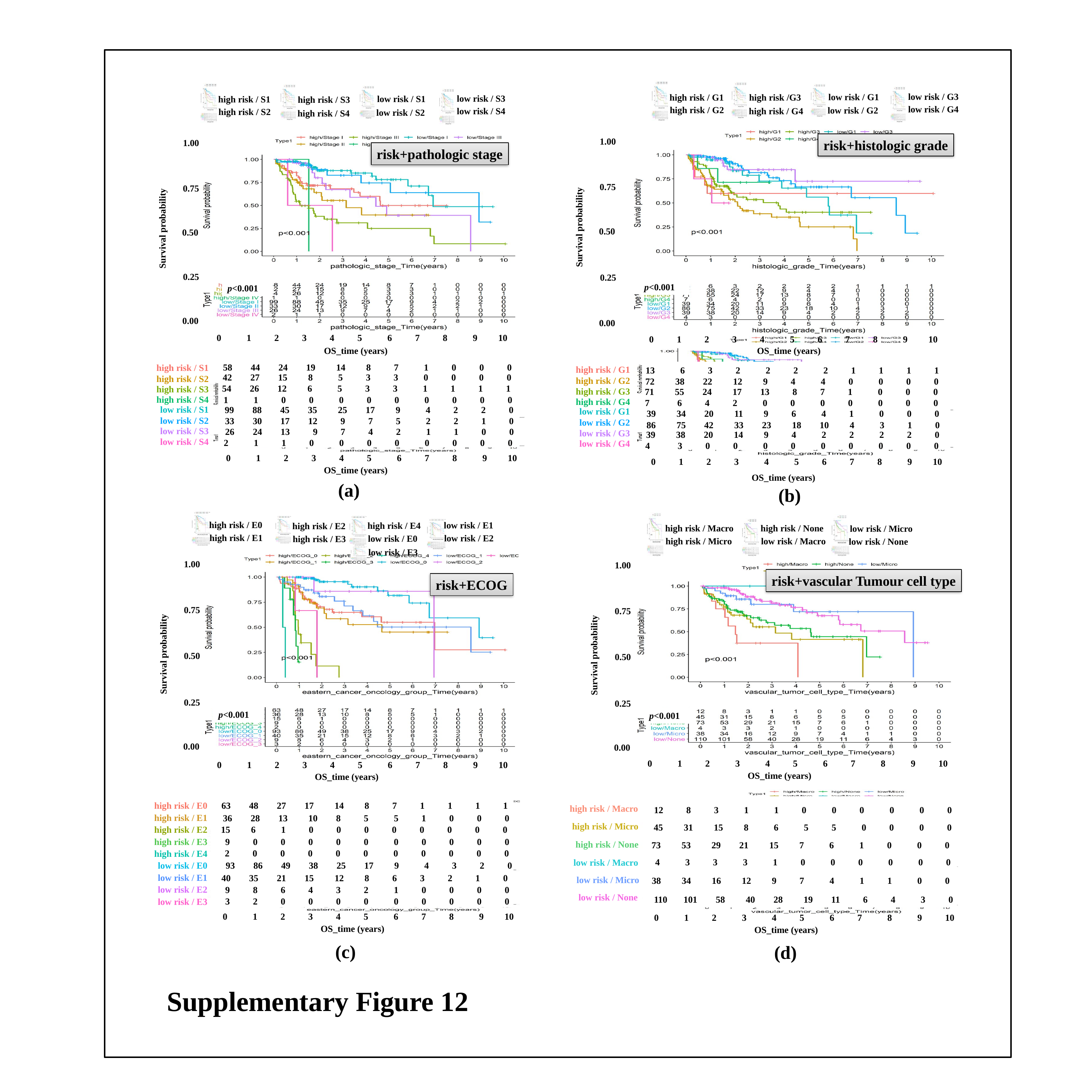

high risk / G1
high risk /G3
high risk / G2
high risk / G4
low risk / G3
low risk / G1
low risk / G4
low risk / G2
high risk / S1
high risk / S3
high risk / S2
high risk / S4
low risk / S3
low risk / S1
low risk / S4
low risk / S2
1.00
0.75
Survival probability
0.50
0.25
p<0.001
0.00
risk+histologic grade
1.00
0.75
Survival probability
0.50
0.25
p<0.001
0.00
risk+pathologic stage
0 1 2 3 4 5 6 7 8 9 10
OS_time (years)
58 44 24 19 14 8 7 1 0 0 0
42 27 15 8 5 3 3 0 0 0 0
54 26 12 6 5 3 3 1 1 1 1
1 1 0 0 0 0 0 0 0 0 0
99 88 45 35 25 17 9 4 2 2 0
33 30 17 12 9 7 5 2 2 1 0
26 24 13 9 7 4 2 1 1 0 0
2 1 1 0 0 0 0 0 0 0 0
0 1 2 3 4 5 6 7 8 9 10
OS_time (years)
high risk / S1
high risk / S2
high risk / S3
high risk / S4
low risk / S1
low risk / S2
low risk / S3
low risk / S4
high risk / G1
high risk / G2
high risk / G3
high risk / G4
low risk / G1
low risk / G2
low risk / G3
low risk / G4
13 6 3 2 2 2 2 1 1 1 1
72 38 22 12 9 4 4 0 0 0 0
71 55 24 17 13 8 7 1 0 0 0
 7 6 4 2 0 0 0 0 0 0 0
 86 75 42 33 23 18 10 4 3 1 0
 39 38 20 14 9 4 2 2 2 2 0
 4 3 0 0 0 0 0 0 0 0 0
 39 34 20 11 9 6 4 1 0 0 0
0 1 2 3 4 5 6 7 8 9 10
OS_time (years)
0 1 2 3 4 5 6 7 8 9 10
OS_time (years)
(a)
(b)
high risk / E0
low risk / E1
low risk / E2
high risk / E4
low risk / E0
high risk / E2
high risk / E3
high risk / Macro
high risk / Micro
high risk / None
low risk / Macro
low risk / Micro
high risk / E1
low risk / None
low risk / E3
1.00
0.75
Survival probability
0.50
0.25
p<0.001
0.00
1.00
0.75
Survival probability
0.50
0.25
p<0.001
0.00
risk+vascular Tumour cell type
risk+ECOG
0 1 2 3 4 5 6 7 8 9 10
OS_time (years)
0 1 2 3 4 5 6 7 8 9 10
OS_time (years)
63 48 27 17 14 8 7 1 1 1 1
36 28 13 10 8 5 5 1 0 0 0
15 6 1 0 0 0 0 0 0 0 0
9 0 0 0 0 0 0 0 0 0 0
2 0 0 0 0 0 0 0 0 0 0
93 86 49 38 25 17 9 4 3 2 0
40 35 21 15 12 8 6 3 2 1 0
9 8 6 4 3 2 1 0 0 0 0
3 2 0 0 0 0 0 0 0 0 0
0 1 2 3 4 5 6 7 8 9 10
OS_time (years)
(c)
high risk / E0
high risk / E1
high risk / E2
high risk / E3
high risk / E4
low risk / E0
low risk / E1
low risk / E2
low risk / E3
high risk / Macro
high risk / Micro
high risk / None
low risk / Macro
low risk / Micro
low risk / None
12 8 3 1 1 0 0 0 0 0 0
73 53 29 21 15 7 6 1 0 0 0
4 3 3 3 1 0 0 0 0 0 0
0 1 2 3 4 5 6 7 8 9 10
OS_time (years)
 45 31 15 8 6 5 5 0 0 0 0
 38 34 16 12 9 7 4 1 1 0 0
 110 101 58 40 28 19 11 6 4 3 0
Supplementary Figure 12
(d)
