# Supplementary material for: Identifying a Novel Endoplasmic Reticulum-Related Prognostic Model for Hepatocellular Carcinomas
Source: Oxid Med Cell Longev. 2022 Jul 22;2022:8248355. doi: 10.1155/2022/8248355 (PMC9338738; doi:10.1155/2022/8248355)
Supplement: Supplementary 1 — Supplementary Figure 1: univariate Cox regression analyses of TCGA-LIHC and GSE14520. We conducted univariate Cox regression analyses to identify a set of HCC prognosis-related candidate genes for TCGA-LIHC OS (a), GSE14520 OS (b), and RFS (c). Supplementary Figure 2: validation analysis of the Lasso regression model. Based on the risk scores of the Lasso regression model, we divided the HCC patients of GSE14520 into high- and low-risk groups. The corresponding heatmaps (a), risk profiles (b), survival status maps (c), survival curves of OS (d), and RFS (e) are shown. Supplementary Figure 3: heatmap for the hub gene expression and clinical traits of HCC patients within TCGA-LIHC cohort. Supplementary Figure 4: heatmap for the hub gene expression and clinical traits of HCC patients within the GSE14520 cohort. Supplementary Figure 5: correlations between the continuous variable index of clinical traits and high/low risk. The differences in the continuous variable index for TCGA cohorts between the high and low groups were analysed by the wilcox.test: height (a), weight (b), BMI (c), creatinine (d), fetoprotein (e), albumin (f), platelet count (g), and prothrombin time (h). Supplementary Figure 6: correlation analysis between hub gene expression and the factors of pathological stage and age or sex. We combined the expression matrix and clinical information of five hub genes from TCGA-LIHC and GSE14520 cohorts and analysed the expression characteristics for the different pathological stages (a, d) and age (b, e), or sex (c, f), using kruskal.test or wilcox.test. ∗p < 0.05, ∗∗p < 0.01, ∗∗∗p < 0.001. Supplementary Figure 7: correlation analysis between hub gene expression and pathological T/N/M. The expression differences in the five hub genes in the different pathological T/N/M groups were analysed by the kruskal.test, followed by the wilcox.test for TCGA cohort. (a) FMO3; (b) KIF2C; (c) KPNA2; (d) LPCAT1; (e) SPP1. Supplementary Figure 8: correlation analysis between hu [file 8248355.f1.zip › Figure S3.pptx]

## Slide 1
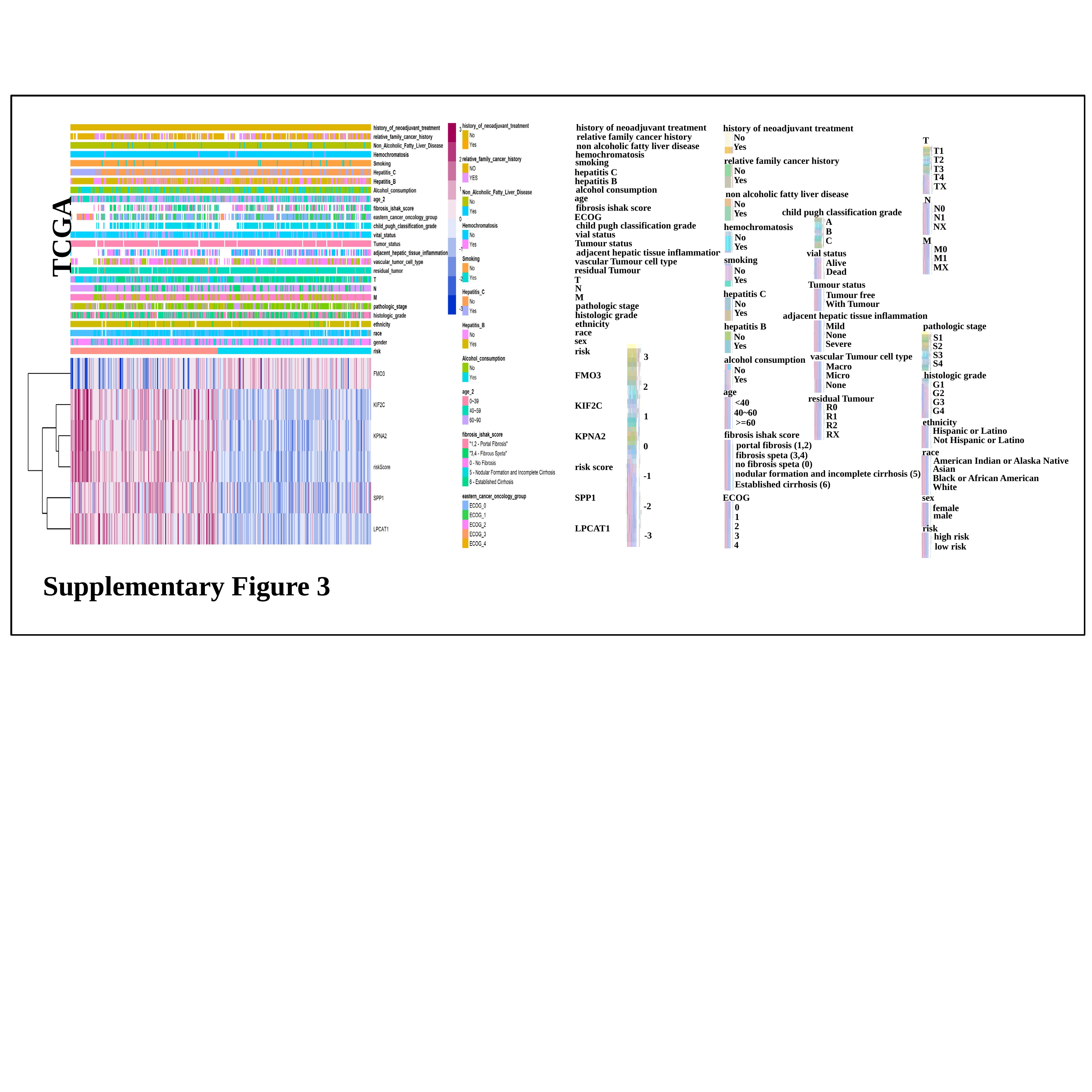

history of neoadjuvant treatment
history of neoadjuvant treatment
relative family cancer history
No
Yes
T
non alcoholic fatty liver disease
T1
T2
T3
hemochromatosis
relative family cancer history
smoking
No
Yes
hepatitis C
T4
hepatitis B
TX
alcohol consumption
non alcoholic fatty liver disease
age
N
No
Yes
fibrosis ishak score
N0
N1
NX
child pugh classification grade
ECOG
A
child pugh classification grade
TCGA
hemochromatosis
B
vial status
No
Yes
M
C
Tumour status
M0
M1
MX
adjacent hepatic tissue inflammation
vial status
smoking
vascular Tumour cell type
Alive
residual Tumour
No
Yes
Dead
T
Tumour status
N
hepatitis C
Tumour free
M
No
Yes
With Tumour
pathologic stage
histologic grade
adjacent hepatic tissue inflammation
ethnicity
Mild
pathologic stage
S1
S2
S3
S4
histologic grade
G1
G2
G3
G4
ethnicity
Hispanic or Latino
Not Hispanic or Latino
race
American Indian or Alaska Native
Asian
Black or African American
White
sex
female
male
risk
high risk
low risk
hepatitis B
race
None
No
Yes
sex
Severe
risk
3
2
1
0
-1
-2
-3
vascular Tumour cell type
alcohol consumption
Macro
No
Yes
FMO3
Micro
None
age
residual Tumour
<40
KIF2C
R0
40~60
R1
>=60
R2
RX
fibrosis ishak score
KPNA2
portal fibrosis (1,2)
fibrosis speta (3,4)
no fibrosis speta (0)
risk score
nodular formation and incomplete cirrhosis (5)
Established cirrhosis (6)
ECOG
SPP1
0
1
2
LPCAT1
3
4
Supplementary Figure 3
