# Supplementary material for: Identifying a Novel Endoplasmic Reticulum-Related Prognostic Model for Hepatocellular Carcinomas
Source: Oxid Med Cell Longev. 2022 Jul 22;2022:8248355. doi: 10.1155/2022/8248355 (PMC9338738; doi:10.1155/2022/8248355)
Supplement: Supplementary 1 — Supplementary Figure 1: univariate Cox regression analyses of TCGA-LIHC and GSE14520. We conducted univariate Cox regression analyses to identify a set of HCC prognosis-related candidate genes for TCGA-LIHC OS (a), GSE14520 OS (b), and RFS (c). Supplementary Figure 2: validation analysis of the Lasso regression model. Based on the risk scores of the Lasso regression model, we divided the HCC patients of GSE14520 into high- and low-risk groups. The corresponding heatmaps (a), risk profiles (b), survival status maps (c), survival curves of OS (d), and RFS (e) are shown. Supplementary Figure 3: heatmap for the hub gene expression and clinical traits of HCC patients within TCGA-LIHC cohort. Supplementary Figure 4: heatmap for the hub gene expression and clinical traits of HCC patients within the GSE14520 cohort. Supplementary Figure 5: correlations between the continuous variable index of clinical traits and high/low risk. The differences in the continuous variable index for TCGA cohorts between the high and low groups were analysed by the wilcox.test: height (a), weight (b), BMI (c), creatinine (d), fetoprotein (e), albumin (f), platelet count (g), and prothrombin time (h). Supplementary Figure 6: correlation analysis between hub gene expression and the factors of pathological stage and age or sex. We combined the expression matrix and clinical information of five hub genes from TCGA-LIHC and GSE14520 cohorts and analysed the expression characteristics for the different pathological stages (a, d) and age (b, e), or sex (c, f), using kruskal.test or wilcox.test. ∗p < 0.05, ∗∗p < 0.01, ∗∗∗p < 0.001. Supplementary Figure 7: correlation analysis between hub gene expression and pathological T/N/M. The expression differences in the five hub genes in the different pathological T/N/M groups were analysed by the kruskal.test, followed by the wilcox.test for TCGA cohort. (a) FMO3; (b) KIF2C; (c) KPNA2; (d) LPCAT1; (e) SPP1. Supplementary Figure 8: correlation analysis between hu [file 8248355.f1.zip › Figure S5.pptx]

## Slide 1
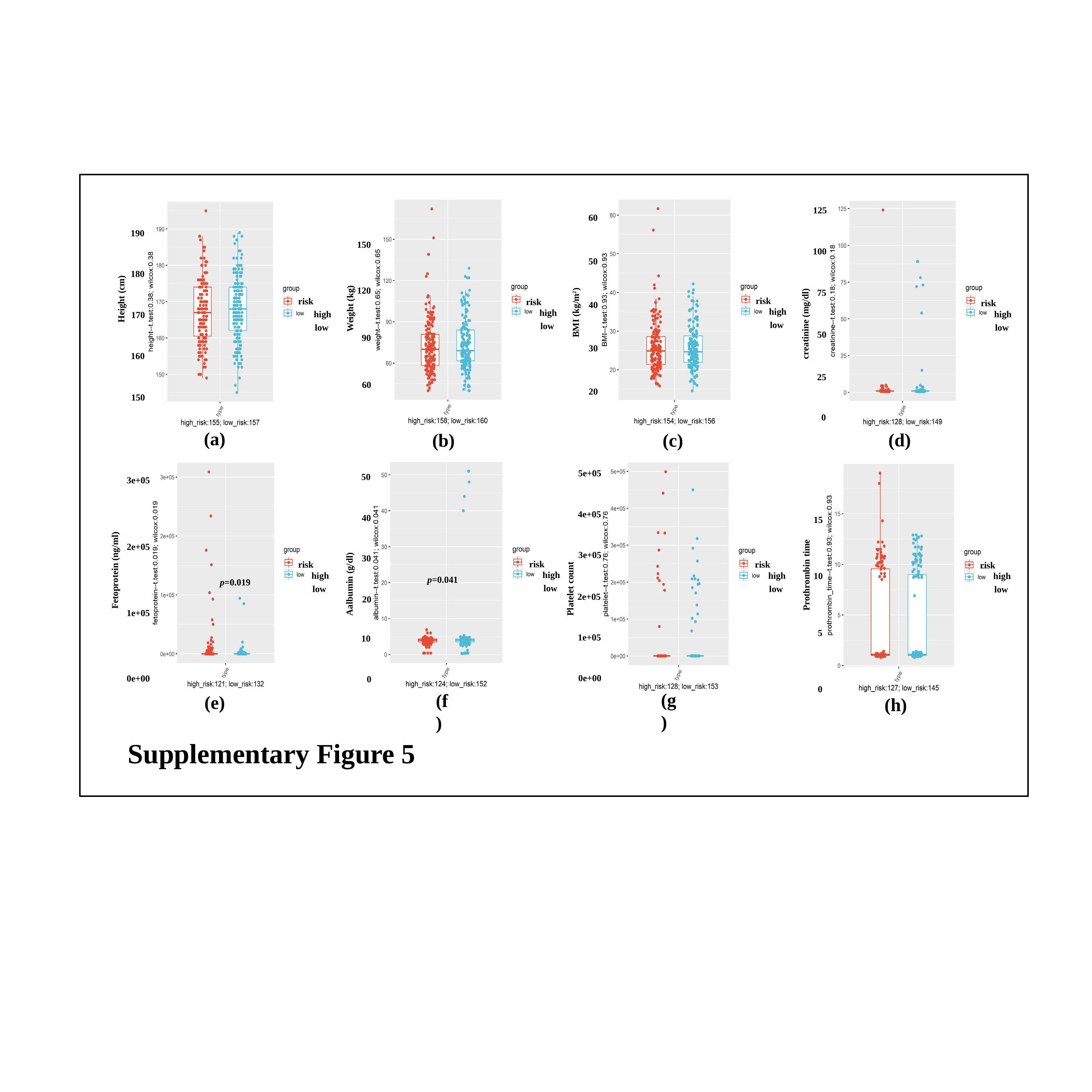

60
50
risk
high
low
40
BMI (kg/m2)
30
20
(c)
125
100
75
risk
high
low
creatinine (mg/dl)
50
25
0
(d)
150
120
Weight (kg)
90
60
(b)
190
180
Height (cm)
risk
high
170
low
160
150
risk
high
low
(a)
risk
high
low
risk
high
low
Prothrombin time
Platelet count
(g)
(h)
5e+05
50
3e+05
4e+05
40
15
2e+05
3e+05
30
risk
high
low
risk
high
low
Fetoprotein (ng/ml)
Aalbumin (g/dl)
10
p=0.019
2e+05
20
1e+05
5
1e+05
10
0e+00
0e+00
0
0
(f)
(e)
Supplementary Figure 5
p=0.041
