# Supplementary material for: Identifying a Novel Endoplasmic Reticulum-Related Prognostic Model for Hepatocellular Carcinomas
Source: Oxid Med Cell Longev. 2022 Jul 22;2022:8248355. doi: 10.1155/2022/8248355 (PMC9338738; doi:10.1155/2022/8248355)
Supplement: Supplementary 1 — Supplementary Figure 1: univariate Cox regression analyses of TCGA-LIHC and GSE14520. We conducted univariate Cox regression analyses to identify a set of HCC prognosis-related candidate genes for TCGA-LIHC OS (a), GSE14520 OS (b), and RFS (c). Supplementary Figure 2: validation analysis of the Lasso regression model. Based on the risk scores of the Lasso regression model, we divided the HCC patients of GSE14520 into high- and low-risk groups. The corresponding heatmaps (a), risk profiles (b), survival status maps (c), survival curves of OS (d), and RFS (e) are shown. Supplementary Figure 3: heatmap for the hub gene expression and clinical traits of HCC patients within TCGA-LIHC cohort. Supplementary Figure 4: heatmap for the hub gene expression and clinical traits of HCC patients within the GSE14520 cohort. Supplementary Figure 5: correlations between the continuous variable index of clinical traits and high/low risk. The differences in the continuous variable index for TCGA cohorts between the high and low groups were analysed by the wilcox.test: height (a), weight (b), BMI (c), creatinine (d), fetoprotein (e), albumin (f), platelet count (g), and prothrombin time (h). Supplementary Figure 6: correlation analysis between hub gene expression and the factors of pathological stage and age or sex. We combined the expression matrix and clinical information of five hub genes from TCGA-LIHC and GSE14520 cohorts and analysed the expression characteristics for the different pathological stages (a, d) and age (b, e), or sex (c, f), using kruskal.test or wilcox.test. ∗p < 0.05, ∗∗p < 0.01, ∗∗∗p < 0.001. Supplementary Figure 7: correlation analysis between hub gene expression and pathological T/N/M. The expression differences in the five hub genes in the different pathological T/N/M groups were analysed by the kruskal.test, followed by the wilcox.test for TCGA cohort. (a) FMO3; (b) KIF2C; (c) KPNA2; (d) LPCAT1; (e) SPP1. Supplementary Figure 8: correlation analysis between hu [file 8248355.f1.zip › Figure S7.pptx]

## Slide 1
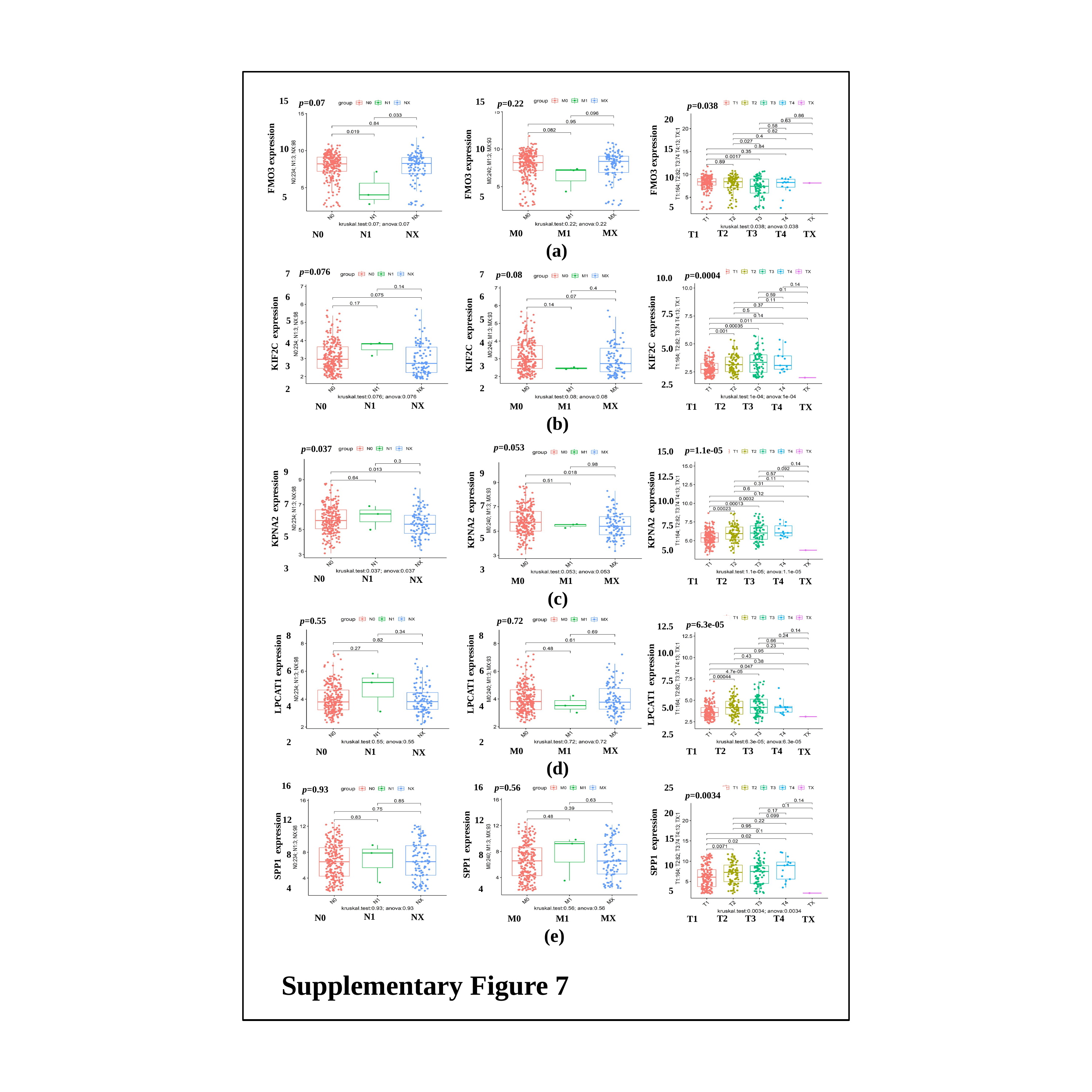

15
15
p=0.07
p=0.22
p=0.038
20
10
15
10
FMO3 expression
FMO3 expression
FMO3 expression
10
5
5
5
MX
M0
M1
T2
T3
T4
TX
N1
T1
NX
N0
(a)
p=0.076
7
7
p=0.08
p=0.0004
10.0
6
6
7.5
5
5
KIF2C expression
KIF2C expression
KIF2C expression
4
4
5.0
3
3
2.5
2
2
N1
NX
MX
M0
M1
N0
T2
T3
T1
T4
TX
(b)
p=0.053
p=0.037
p=1.1e-05
15.0
KPNA2 expression
7.5
5.0
9
7
KPNA2 expression
5
3
9
7
KPNA2 expression
5
3
12.5
10.0
N1
N0
NX
MX
M0
M1
T2
T3
T4
TX
T1
(c)
p=0.55
p=0.72
p=6.3e-05
12.5
10.0
7.5
LPCAT1 expression
8
6
LPCAT1 expression
4
2
8
6
LPCAT1 expression
4
2
5.0
2.5
MX
M0
M1
T2
T3
T4
T1
N1
N0
TX
NX
(d)
16
16
p=0.56
25
p=0.93
p=0.0034
20
12
12
15
SPP1 expression
SPP1 expression
SPP1 expression
8
8
10
4
4
5
N1
NX
N0
MX
M0
M1
T2
T3
T4
T1
TX
(e)
Supplementary Figure 7
