# Supplementary material for: Identifying a Novel Endoplasmic Reticulum-Related Prognostic Model for Hepatocellular Carcinomas
Source: Oxid Med Cell Longev. 2022 Jul 22;2022:8248355. doi: 10.1155/2022/8248355 (PMC9338738; doi:10.1155/2022/8248355)
Supplement: Supplementary 1 — Supplementary Figure 1: univariate Cox regression analyses of TCGA-LIHC and GSE14520. We conducted univariate Cox regression analyses to identify a set of HCC prognosis-related candidate genes for TCGA-LIHC OS (a), GSE14520 OS (b), and RFS (c). Supplementary Figure 2: validation analysis of the Lasso regression model. Based on the risk scores of the Lasso regression model, we divided the HCC patients of GSE14520 into high- and low-risk groups. The corresponding heatmaps (a), risk profiles (b), survival status maps (c), survival curves of OS (d), and RFS (e) are shown. Supplementary Figure 3: heatmap for the hub gene expression and clinical traits of HCC patients within TCGA-LIHC cohort. Supplementary Figure 4: heatmap for the hub gene expression and clinical traits of HCC patients within the GSE14520 cohort. Supplementary Figure 5: correlations between the continuous variable index of clinical traits and high/low risk. The differences in the continuous variable index for TCGA cohorts between the high and low groups were analysed by the wilcox.test: height (a), weight (b), BMI (c), creatinine (d), fetoprotein (e), albumin (f), platelet count (g), and prothrombin time (h). Supplementary Figure 6: correlation analysis between hub gene expression and the factors of pathological stage and age or sex. We combined the expression matrix and clinical information of five hub genes from TCGA-LIHC and GSE14520 cohorts and analysed the expression characteristics for the different pathological stages (a, d) and age (b, e), or sex (c, f), using kruskal.test or wilcox.test. ∗p < 0.05, ∗∗p < 0.01, ∗∗∗p < 0.001. Supplementary Figure 7: correlation analysis between hub gene expression and pathological T/N/M. The expression differences in the five hub genes in the different pathological T/N/M groups were analysed by the kruskal.test, followed by the wilcox.test for TCGA cohort. (a) FMO3; (b) KIF2C; (c) KPNA2; (d) LPCAT1; (e) SPP1. Supplementary Figure 8: correlation analysis between hu [file 8248355.f1.zip › Figure S9.pptx]

## Slide 1
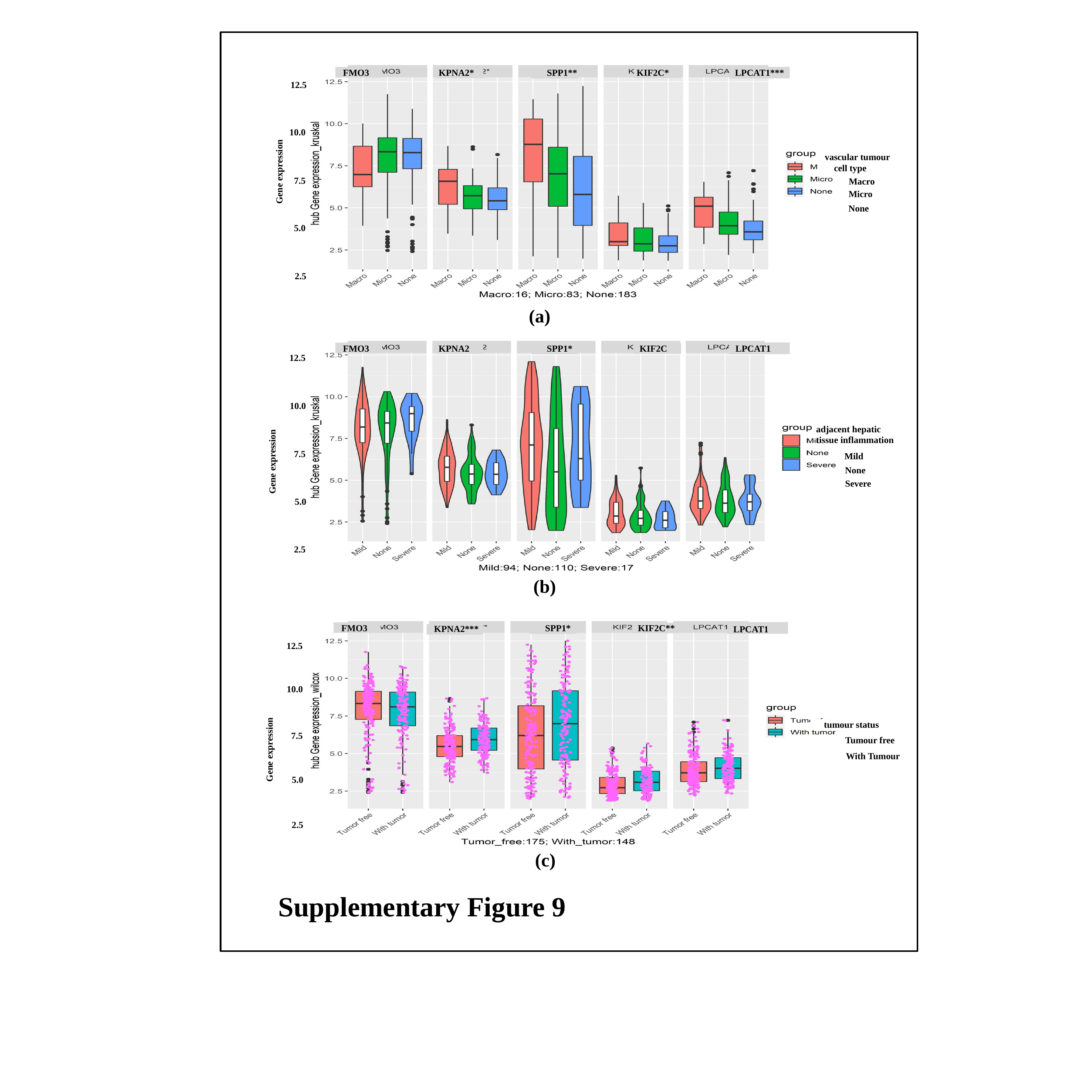

Supplementary Figure 9
FMO3
KPNA2*
SPP1**
KIF2C*
LPCAT1***
12.5
10.0
vascular tumour
 cell type
Gene expression
7.5
Macro
Micro
None
5.0
2.5
(a)
FMO3
KPNA2
SPP1*
KIF2C
LPCAT1
12.5
10.0
7.5
Gene expression
5.0
2.5
adjacent hepatic
tissue inflammation
Mild
None
Severe
(b)
FMO3
SPP1*
KIF2C**
KPNA2***
LPCAT1
12.5
10.0
7.5
Gene expression
5.0
2.5
tumour status
Tumour free
With Tumour
(c)
