# Supplementary material for: Identifying a Novel Endoplasmic Reticulum-Related Prognostic Model for Hepatocellular Carcinomas
Source: Oxid Med Cell Longev. 2022 Jul 22;2022:8248355. doi: 10.1155/2022/8248355 (PMC9338738; doi:10.1155/2022/8248355)
Supplement: Supplementary 2 — Table S1: clinical characteristics of HCC cases for TCGA-LIHC. Table S2: clinical characteristics of HCC cases for GSE14520. [file 8248355.f2.zip › Table S1.docx]

**Supplementary Table 1.** **Clinical characteristics of HCC cases for TCGA-LIHC.**

| **characteristics** | **group** | **number** | **%** |
| --- | --- | --- | --- |
| **sex** | female | 105 | 31.2 |
|  | male | 231 | 68.8 |
| **race** | American Indian or Alaska Native | 1 | 0.3 |
|  | Asian | 149 | 45.7 |
|  | Black or African American | 14 | 4.3 |
|  | White | 162 | 49.7 |
| **ethnicity** | Hispanic or Latino | 17 | 5.3 |
|  | not Hispanic or Latino | 305 | 94.7 |
| **histologic grade** | G1 | 52 | 15.7 |
|  | G2 | 158 | 47.7 |
|  | G3 | 110 | 33.2 |
|  | G4 | 11 | 3.3 |
| **pathologic stage** | stage 1 | 157 | 49.8 |
|  | stage 2 | 75 | 23.8 |
|  | stage 3 | 80 | 25.4 |
|  | stage 4 | 3 | 1 |
| **M** | M0 | 240 | 71.4 |
|  | M1 | 3 | 0.9 |
|  | MX | 93 | 27.7 |
| **N** | N0 | 234 | 69.9 |
|  | N1 | 3 | 0.9 |
|  | NX | 98 | 29.3 |
| **T** | T1 | 164 | 49.1 |
|  | T2 | 82 | 24.6 |
|  | T3 | 74 | 22.2 |
|  | T4 | 13 | 3.9 |
|  | TX | 1 | 0.3 |
| **residual tumour** | R0 | 299 | 90.9 |
|  | R1 | 14 | 4.3 |
|  | R2 | 1 | 0.3 |
|  | RX | 15 | 4.6 |
| **vascular tumour cell type** | Macro | 16 | 5.7 |
|  | Micro | 83 | 29.4 |
|  | None | 183 | 64.9 |
| **adjacent hepatic tissue inflammation** | Mild | 94 | 42.5 |
|  | None | 110 | 49.8 |
|  | Severe | 17 | 7.7 |
| **tumour status** | tumour free | 175 | 54.2 |
|  | with tumour | 148 | 45.8 |
| **vital status** | alive | 255 | 75.9 |
|  | dead | 81 | 24.1 |
| **Child pugh classification grade** | A | 202 | 90.6 |
|  | B | 20 | 9 |
|  | C | 1 | 0.4 |
| **ECOG** | ECOG_0 | 156 | 57.8 |
|  | ECOG_1 | 76 | 28.1 |
|  | ECOG_2 | 24 | 8.9 |
|  | ECOG_3 | 12 | 4.4 |
|  | ECOG_4 | 2 | 0.7 |
| **fibrosis ishak score** | 0 - No Fibrosis | 70 | 35.2 |
|  | 1,2 - Portal Fibrosis | 30 | 15.1 |
|  | 3,4 - Fibrous Speta | 24 | 12.1 |
|  | 5 - Nodular Formation and Incomplete Cirrhosis | 8 | 4 |
|  | 6 - Established Cirrhosis | 67 | 33.7 |
| **age** | 0~39 | 27 | 8 |
|  | 40~59 | 128 | 38.1 |
|  | 60~90 | 181 | 53.9 |
| **alcohol consumption** | no | 223 | 66.4 |
|  | yes | 113 | 33.6 |
| **hepatitis B** | no | 144 | 42.9 |
|  | yes | 192 | 57.1 |
| **hepatitis C** | no | 208 | 61.9 |
|  | yes | 128 | 38.1 |
| **smoking** | no | 320 | 95.2 |
|  | yes | 16 | 4.8 |
| **hemochromatosis** | no | 329 | 97.9 |
|  | yes | 7 | 2.1 |
| **nonalcoholic fatty liver disease** | no | 318 | 94.6 |
|  | yes | 18 | 5.4 |
| **relative family cancer history** | no | 191 | 65.4 |
|  | yes | 101 | 34.6 |
| **history of neoadjuvant treatment** | no | 334 | 99.4 |
|  | yes | 2 | 0.6 |

ECOG, Eastern cancer oncology group
