# Supplementary material for: Identifying a Novel Endoplasmic Reticulum-Related Prognostic Model for Hepatocellular Carcinomas
Source: Oxid Med Cell Longev. 2022 Jul 22;2022:8248355. doi: 10.1155/2022/8248355 (PMC9338738; doi:10.1155/2022/8248355)
Supplement: Supplementary 2 — Table S1: clinical characteristics of HCC cases for TCGA-LIHC. Table S2: clinical characteristics of HCC cases for GSE14520. [file 8248355.f2.zip › Table S2 .docx]

**Supplementary Table 2.** **Clinical characteristics of HCC cases for GSE14520.**

| **Characteristics** | **group** | **number** | **%** |
| --- | --- | --- | --- |
| **sex** | female | 31 | 12.8 |
|  | male | 211 | 87.2 |
| **age** | 0~39 | 34 | 14.2 |
|  | 40~59 | 157 | 65.7 |
|  | 60~90 | 48 | 20.1 |
| **hepatitis B virus** | active viral replication chronic carrier | 58 | 26.2 |
|  | chronic carrier | 157 | 71 |
|  | normal | 6 | 2.7 |
| **multinodular** | no | 187 | 78.2 |
|  | yes | 52 | 21.8 |
| **cirrhosis** | no | 19 | 7.9 |
|  | yes | 220 | 92.1 |
| **pathologic stage** | stage 1 | 93 | 41.9 |
|  | stage 2 | 78 | 35.1 |
|  | stage 3 | 51 | 23 |
| **BCLC_staging** | 0 | 20 | 9 |
|  | A | 149 | 67.1 |
|  | B | 24 | 10.8 |
|  | C | 29 | 13.1 |
| **CLIP_staging** | CLIP_0 | 97 | 43.7 |
|  | CLIP_1 | 77 | 34.7 |
|  | CLIP_2 | 35 | 15.8 |
|  | CLIP_3_4_5 | 13 | 5.9 |
| **ALT** | high | 99 | 41.4 |
|  | low | 140 | 58.6 |
| **main tumour size** | large | 86 | 36.1 |
|  | small | 152 | 63.9 |
| **AFP** | high | 108 | 46 |
|  | low | 127 | 54 |

BCLC, Barcelona clinic liver cancer; CLIP, cancer of the liver Italian program;

ALT, alanine aminotransferase; AFP, alpha-fetoprotein
